# Peripheral blood lymphocytes differentiation patterns in responses / outcomes to immune checkpoint blockade therapies in non-small cell lung cancer: a retrospective study

**DOI:** 10.1186/s12885-023-10502-4

**Published:** 2023-01-25

**Authors:** Xiaoyue Du, Shaodi Wen, Run Shi, Jingwei Xia, Ruotong Wang, Yihan Zhang, Banzhou Pan, Xiaoliu Wu, Wei Zhu, Jifeng Feng, Xin Wang, Bo Shen

**Affiliations:** 1 Department of Oncology, The Affiliated Cancer Hospital of Nanjing Medical University, Jiangsu Cancer Hospital, Jiangsu Institute of Cancer Research, Nanjing, China; 2 Department of Oncology, The First Affiliated Hospital of Nanjing Medical University, Nanjing, China; 3 Flow Cytometry Core Facility, The Affiliated Cancer Hospital of Nanjing Medical University, Jiangsu Cancer Hospital, Jiangsu Institute of Cancer Research, Nanjing, China; 4 School of Medicine, Jiangsu University, Zhenjiang, China

**Keywords:** Lymphocytes differentiation, Immunotherapy, Non-small cell lung cancer, α-PD-1 / PD-L1, Nomogram

## Abstract

**Objectives:**

Programmed Cell Death-1/ Programmed Death-ligand 1 (PD-1 / PD-L1) inhibitor therapies targeting immunocytes induce persistent tumor remission in various cancers. However, the appropriate biomarkers for the therapeutic efficacy of PD-L1 and PD-1 blockade remain elusive.

**Materials and methods:**

For a comprehensive analysis of peri-treatment lymphocyte differentiation, in the current study, we enrolled 146 non-small cell lung cancer patients who received α-PD-1 therapies for exploring the peripheral blood lymphocyte differentiation pattern at baseline and post-treatment (dynamic changes) by flow cytometry.

**Results:**

At baseline, CD4^+^ / CD8^+^ T cell ratio predicts good responses and outcomes, but activated T cell and cytotoxic T cell counts predict poor responses and outcomes. And for dynamic changes, after 6 weeks of immune checkpoint blockade (ICB) treatment, compared with baseline level, the elevation of total T and B cell counts indicate poor responses, and total T and T_H_ cell counts indicate poor prognosis while activated T cell predicts good prognosis. And after 12 weeks, elevated total lymphocyte, cytotoxic T cell counts, and decreased total T cell counts and CD4^+^ / CD8^+^ T cell ratio predict good responses / outcomes. Our clinical predicting model shows good performance in predicting ICB treatment responses / outcomes.

**Conclusion:**

Patients with favorable clinical responses / outcomes have distinctive peripheral blood immunocyte differentiation characteristics, indicating the potential of utilizing the peripheral immunocyte differentiation patterns for predicting ICB responses / outcomes.

**Supplementary Information:**

The online version contains supplementary material available at 10.1186/s12885-023-10502-4.

## Introduction

The immune system was explored as a complex stable network. In healthy conditions, the immune checkpoints play vital roles in protecting from autoimmune diseases [1]. In malignant conditions, tumors may exploit peripheral immune tolerance (especially against cytotoxic T cells) for tumorigenesis by orchestrating these immune checkpoints [2]. As an important immune checkpoint axis, the Programmed Cell Death-1/ Programmed Death-ligand 1 (PD-1 / PD-L1) axis was first reported in autoimmune-induced inflammation, but now this axis was more famous for its role in suppressing anti-tumor immunity [3].

Malignant cells usually acquire immune tolerance by following mechanisms: 1) Suppress immunogenicity by down-regulating tumor-specific or related antigen, and / or impairing antigen presentation capability (e.g., the dysfunction of major histocompatibility complex class-I antigen presentation system) [4]. 2) Up-regulating the immunosuppressive ligand on the cell surface (e.g., Fas ligand, CD44, PD-L1, etc.) [5, 6]. 3) Remodeling microenvironment secretome, which not only promotes host immune tolerance but also might enhance tumor stemness / proliferation (e.g., regulating granulocyte colony-stimulating factor, IL-10, and IL-6, etc.) [7, 8]. 4) Recruiting immunosuppressive cells in the microenvironment (e.g., myeloid-derived suppressor cells, regulatory T cells, etc.) [9, 10]. These mechanisms together undermine the balance between pro-and anti-tumor immune responses and contribute to tumor immune escape.

Currently, α-PD-1 / PD-L1 aiming at switching off immune checkpoint is the most popular immune checkpoint blockade strategy. PD-1 also known as CD279 is a receptor mainly expressed on the surface of T and pro-B cells, and two ligands could bind to this receptor, PD-L1 and PD-L2 [11]. Originally, several lines of evidence suggested that the PD-1 / PD-L1 axis negatively regulates immune responses, in mice models PD-1 knockout lead to severe autoimmune diseases [12, 13]. And recently, more evidence revealed its role in evading immune surveillance and suppressing anti-tumor immunity, highlighting this axis as a target for immunotherapy.

Clinically, α-PD-1 / PD-L1 cancer immunotherapy continues to progress at a fast speed, and therapeutic strategies and pharmaceutic development are evolving rapidly to maximize patient benefit. In several solid tumors, especially lung cancer, α-PD-1 / PD-L1 immunotherapy has already been adopted in the first-line approaches for late-stage, adjuvant, and neoadjuvant cancer treatments [14–16]. But only a fraction of patients with solid tumors responds well to α-PD-1 / PD-L1 therapy (around 20–40%, depending on cancer types) [2]. So, why some patients don’t respond to α-PD-1 / PD-L1 immunotherapy is one of the major questions in the field. Currently, biomarkers, such as neutrophil-to-lymphocyte ratio, gut microbiota, tumor-infiltrating lymphocytes, etc., are used for predicting immunotherapy’s efficacy in non-small cell lung cancer (NSCLC) [17, 18], and PD-L1 and tumor mutation burden (TMB) remain the most widely used biomarkers approved by the Food and Drug Administration (FDA). Of note, recently concerns were raised about the adequacy of traditional markers / indicators for immune checkpoint blockade (ICB) treatment, such as microenvironment PD-L1 level and TMB [19, 20]. Hence, discriminating potential α-PD-1 / PD-L1 immunotherapy beneficiaries with adequate biomarkers still remains an urgent priority [21].

Considering α-PD-1 / PD-L1 immunotherapy targets immunocytes and is designed to shift the immune balance towards anti-tumor response, the attempt of monitoring dynamic differentiation changes of immunocytes for evaluating neo indicators for α-PD-1 / PD-L1 immunotherapy is reasonable. Of note, different from traditional tissue-based methods (for evaluating PD-L1 or TMB level), a milliliter level blood-based method evaluating the differentiation status of immunocytes provides a flexible alternative. We introduced flow cytometry as an appropriate method in current immune-related research. Flow cytometry (FCM) is a laser fluorescence-based technique used to detect and analyze the chemical / biological and optical characteristics of cells and particles. In basic research / clinical practice, compared with traditional protein detection approaches (such as immunohistochemistry and immunoblot), FCM featured multiplex and high sensitivity. In the medical laboratory, FCM had been wielded adopted as a powerful tool for immunology-related measurement in hematopoietic malignancies, autoimmune diseases, and allograft transplants [22–24].

In the current study, we evaluated the potential of monitoring the differentiation of immunocytes in peripheral blood as predictors / indicators for α-PD-1 therapy. We reported several interesting lymphocytes’ differentiation pattern and clinical parameters correlates with ICB response / outcomes.

## Material and methods

### Study design

Patients were enrolled from conventional treatments or clinical trials at the Affiliated Cancer Hospital of Nanjing Medical University. For inclusion criteria: patients were diagnosed with late-stage NSCLC, with at least one measurable lesion (according to Immune-related Response Evaluation Criteria in Solid Tumors), without a history of α-PD-1 / PD-L1 treatment, and with peripheral blood lymphocytes flow cytometry data and tumor markers (carcinoembryonic antigen, carbohydrate antigen 125, carcinoembryonic antigen199, neuron-specific enolase) for the following 3 time points: baseline, 6 and 12 weeks after treatment. The following patients were excluded: (I) with comorbidities (e.g., heart failure, kidney and/or liver failure, severe diabetes mellitus); (II) with severe mental disorders; (III) with a history of other malignancies; and (IV) special populations (e.g., pregnant and lactating women). 146 patients received α-PD-1 treatment regimens (Pembrolizumab / Sintilimab / Toripalimab / Camrelizumab / Tislelizumab) from August 2018 to May 2021 were enrolled, the last follow-up time was December 31, 2021. All enrolled patients received α-PD-1 intravenously once every 3 weeks until disease progression or unacceptable toxicity, combination treatment regimens are determined by clinicians based on the patient’s condition. Detailed study process and patient characteristics are shown in Table 1, Supplement Table 1 and Supplement Fig. 1A and B. This study was approved by the Institutional Review Board of Jiangsu Cancer Hospital.

**Table 1:** Patients’ baseline characteristics

Parameters	Total (*N* = 146)	DCB (*N* = 120)	NDB (*N* = 26)	*P* value
Age(years) ^a^	64(56–69)			0.346
< 64	72 (49.3)	57 (47.5)	15 (57.7)	
≥ 64	74 (50.7)	63 (52.5)	11 (42.3)	
Gender ^b^				
Male	111 (76.0)	93 (77.5)	18 (69.2)	0.371
Female	35 (24.0)	27 (22.5)	8 (30.8)	
Histology ^b^				
Non- Squamous	101 (69.2)	82 (68.3)	19 (73.1)	0.635
Squamous	45 (30.8)	38 (31.7)	7 (26.9)	
Stage ^b^				
IIIB	27 (18.5)	25 (20.8)	2 (7.7)	0.118
IV	119 (81.5)	95 (79.2)	24 (92.3)	
Differentiation ^b^				
Moderate	13 (8.9)	9 (7.5)	4 (15.4)	0.641
Medium-Low	18 (12.3)	15 (12.5)	3 (11.5)	
Low	34 (23.3)	28 (23.3)	6 (23.1)	
NA	81 (55.5)	68 (56.7)	13 (50.0)	
ECOG PS ^b^				
0	23 (15.8)	18 (15.0)	5 (19.2)	0.185
1	108 (74.0)	92 (76.7)	16 (61.5)	
2	15 (10.3)	10 (8.3)	5 (19.2)	
Smoking history ^b^				
Never	62 (42.5)	48 (40.0)	14 (53.8)	0.195
Now/Ever	84 (57.5)	72 (60.0)	12 (46.2)	
Distant metastases ^b^				
No	27 (18.5)	25 (20.8)	2 (7.7)	0.118
Yes	119 (81.5)	95 (79.2)	24 (92.3)	
Driver mutations ^b^				
No	113 (77.4)	96 (80.0)	17 (65.4)	0.106
Yes	33 (22.6)	24 (20.0)	9 (34.6)	
PD-1 inhibitor type ^b^				
Pembrolizumab	47 (32.2)	43 (35.8)	4 (15.4)	0.320
Toripalimab	18 (12.3)	13 (10.8)	5 (19.2)	
Camrelizumab	31 (21.2)	24 (20.0)	7 (26.9)	
Sintilimab	44 (30.1)	35 (29.2)	9 (34.6)	
Tislelizumab	6 (4.1)	5 (4.2)	1 (3.8)	
Combination regimen ^b^				
Monotherapy	25 (17.1)	19 (15.8)	6 (23.1)	0.787
Chemotherapy	89 (61.0)	75 (62.5)	14 (53.8)	
Anti-angiogenic therapy	28 (19.2)	23 (19.2)	5 (19.2)	
Both	4 (2.7)	3 (2.5)	1 (3.8)	
Drug regiment ^b^				
1st line	62 (42.5)	53 (44.2)	9 (34.6)	0.372
≥ 2nd line	84 (57.5)	67 (55.8)	17 (65.4)	
Radiotherapy ^b^				
No	69 (47.3)	53 (44.2)	16 (61.5)	0.108
Yes	77 (52.7)	67 (55.8)	10 (38.5)	

*ECOG PS* Eastern Cooperative Oncology Group Performance Status, *PD-1* Programmed cell death protein 1, *CR* Complete response, *PR* Partial response, *SD* Stable disease, *PD* Progressed disease

^a^ Median and interquartile range (IQR)

^b^ N (%)

**Fig. 1:**
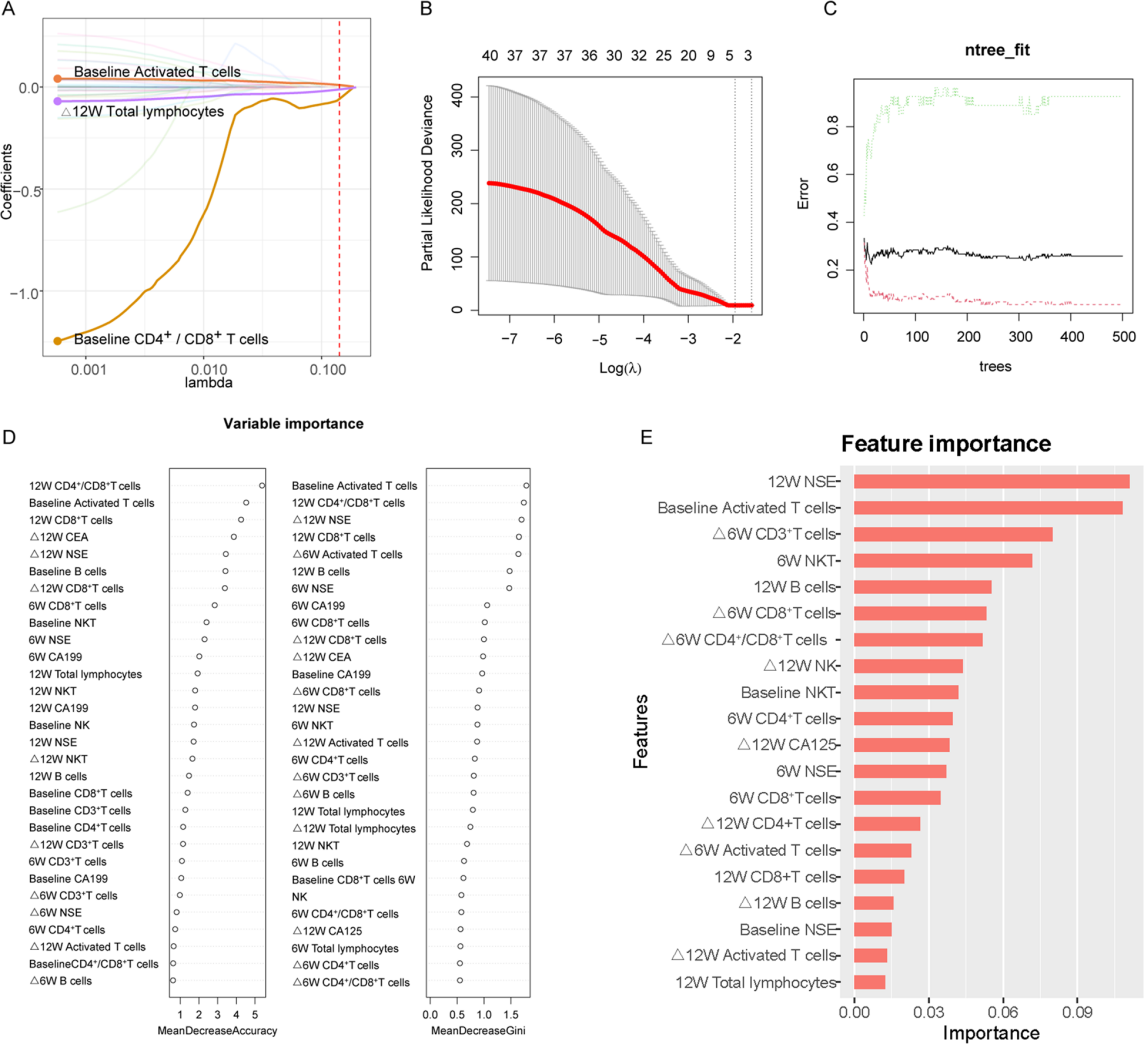
Using machine learning methods to analyze all available data. **A** Distribution of LASSO coefficients for variables. **B** Partial likelihood bias of the LASSO coefficient distribution. The vertical dashed line indicates the minimum partial likelihood deviation. **C** and **D** Random Forest for variable selection. Features identified by the Random Forest model according to the mean decrease in Gini index and Mean Decrease Accuracy for prediction of ICB outcomes. **E** Importance matrix plot of ICB’s predictors in XGBoost model among non-small lung cell cancer patients. NK, natural killer; NKT, natural killer T; CEA, carcinoembryonic antigen; CA125, carbohydrate antigen 125; CA199, carbohydrate antigen 199; NSE, neuron-specific enolase; LASSO, least absolute shrinkage and selection operator; XGBoost, eXtreme Gradient Boosting, RF, random forest

By referring to computed tomography, clinical responses were categorized according to the immune-related response criteria as either complete response (CR), partial response (PR), stable disease (SD), or progressed disease (PD). And during the whole follow-up time, CR / PR / SD lasted > 6 months was defined as durable clinical benefit (DCB), while PD or SD lasted ≤ 6 months was defined as non-durable benefit (NDB). Survival was evaluated by progression-free survival (PFS defined as the time from initial treatment to clinical or imaging progression or death) and overall survival (OS defined as the time from initial treatment to the last follow-up or death).

### Flow cytometry

Peripheral blood mononuclear cells (PBMC) were isolated using Ficoll-Hypaque density gradient centrifugation, and subsequently pre-incubated PBMCs with Fc-block and stain with antibodies to identify total lymphocytes / T and T cell subsets / B cells / Natural killer cells (NK cells). Antibody panels and gating strategies are presented in Supplementary Tables 2 and Supplementary Fig. 1C. Flow analysis was performed on a BD FACS Canto II (BD Biosciences), data were analyzed using FlowJo.

### Statistical analyses

Patients were randomly divided into training set (*n* = 116) and validation set (*n* = 30) according to a ratio of 8:2. And patients’ baseline peripheral blood parameters were categorized by optimal cut-off values (Low / High group), and post-treatment data minus baseline data were defined as dynamic changes.

Variables were selected via integrated analysis of three algorithms consisting of the Least absolute shrinkage and selection operator (LASSO) algorithm with penalty parameter tuning conducted by 10-fold cross-validation, the Random Forest (RF) algorithm searching for lambda with the smallest classification error to determine the variable and adopting the interpretable extreme gradient boosting (XGBoost) algorithm. According to the feature importance ranking, the high-relevance features were found.

In addition to this, Mann-Whitney U test was performed to determine differences between DCB and NDB patients (continuous variables). Chi-square or Fisher’s exact test was used to analyze the association between clinical response and categorical variable, and *p*-values < 0.05 variables were considered statistically significant and continued to be examined through multivariable logistic regression. Survival probabilities were assessed by Kaplan-Meier analysis paired with the Log-rank test or the Cox regression. Nomogram prediction model was constructed using multivariable analysis identified predictive factors. Area under the curve (AUC) and the C-index were used to evaluate the discriminative power of the model, the calibration curve and the decision curve analysis (DCA) were used to evaluate the calibration and clinical effectiveness of the model, respectively.

We calculated the sample size of the multivariable Cox regression model for patients’ overall survival using the previously reported method [25]. Based on the generally accepted rule of thumb of 10 events per variable and the final Cox model containing 2 variables [26], the field size was expected to be 20 events. We used a sample size of at least 108 patients based on an estimated 23% 3-year event rate and a 20% loss-to-review rate among the participants. Besides, we explored the relationship between infiltrating lymphocytes and prognosis in NSCLC patients with The Cancer Genome Atlas Program database (Supplement Fig. 6). All analysis and graphing were powered by FlowJo 10.0 / R studio 4.0.5 / SPSS 26.0 / GraphPad Prism 8.0.

## Results

### Patient characteristics and study design

This study enrolled 146 advanced NSCLC patients treated with α-PD-1 therapy. The median follow-up time, median PFS were 23.3 months (95%CI: 21.8 to 24.7 months), 17.4 months (95%CI: 11.8 to 22.9 months), respectively. And the median OS was not reached yet. Overall, 77/146 patients (52.7%) progressed, and 33/146 (22.6%) patients died during follow-up. According to clinical response, we found that 82.1% patients achieved durable clinical benefit, and 17.8% patients were not. The patients’ characteristics are shown in Table 1. The median age of all patients was 64 years, 76% of patients were male and 42.5% were never-smokers. Almost all patients were Eastern Cooperative Oncology Group performance status (ECOG PS) score 0–1. Most patients had distant metastasis (81.5%) and were in stage IV (81.5%). 22.6% of patients had driver mutations. Therapeutically, 42.5% of patients received α-PD-1 inhibitor as the first line. More than half of patients received chemotherapy combination regimen (82.9%) and ever had radiotherapy during immunotherapy (52.7%). Their detailed peripheral blood parameters are shown in Supplementary Tables 3 and 4.

We introduced the LASSO algorithm (Fig. 1A, B), the RF algorithm (Fig. 1C, D) and XGBoost algorithm for variables selection (Fig. 1E). The variables identified by lasso regression include baseline activate T cell counts, Δ12W total lymphocyte counts and baseline CD4^+^ / CD8^+^ T cell ratio. The importance ranking of the variables determined by the RF and XGBoost algorithms were described in Fig. 1D, E.

### Baseline / dynamic peripheral lymphocytes’ differentiation predicts ICB treatment response

#### Baseline peripheral immunocytes differentiation predicts immunotherapeutic responses

We conducted a univariate analysis to clarify the correlation between peripheral blood immunocytes differentiation and ICB response (Supplementary Table 5). We noticed that before ICB therapy, DCB patients displayed lower percentage of activated T cells, lower level of CEA and higher percentage of CD4^+^ / CD8^+^T cells compared to NDB patients (*p* = 0.006, *p* = 0.009, p = 0.024, respectively). By introducing multivariate logistic regression models (Supplementary Table 6), we found that baseline activated T cells and CEA were independent and effective prognostic factor (*p* = 0.031, OR = 0.066; *p* = 0.013, OR = 0.106, respectively).

#### Dynamic lymphocytes’ differentiation predicts immunotherapeutic responses

We investigated whether dynamic changes (6&12 weeks) of peripheral lymphocytes’ differentiation after ICB treatment could predict patients’ responses and outcomes. We enrolled patients with all three time points (baseline, 6 and 12 weeks) differentiation data. After 6 weeks of ICB treatment, we found that total T cell and B cell counts were able to distinguish DCB or NDB in patients (*p* = 0.024, *p* = 0.026, respectively, Supplementary Table 5). After 12 weeks of ICB treatment, we found that the rise of total lymphocyte and cytotoxic T cells (CTL) counts trends to predict good response to ICB treatment (*p* = 0.000, *p* = 0.026, respectively, Supplementary Table 5). While the elevation of total T cell counts, CD4^+^ / CD8^+^ T cell ratio and CA125 were associated with worse responses (*p* = 0.010, *p* = 0.024, *p* = 0.027, respectively, Supplementary Table 5). As shown in Supplementary Tables 6, the dynamic changes in the percentage of total lymphocytes and the level of CA125 had predictive value for distinguishing DCB or NDB in patients (*p *=0.002, OR = 13.787; *p* =0.027, OR = 0.160, respectively).

**Fig. 2:**
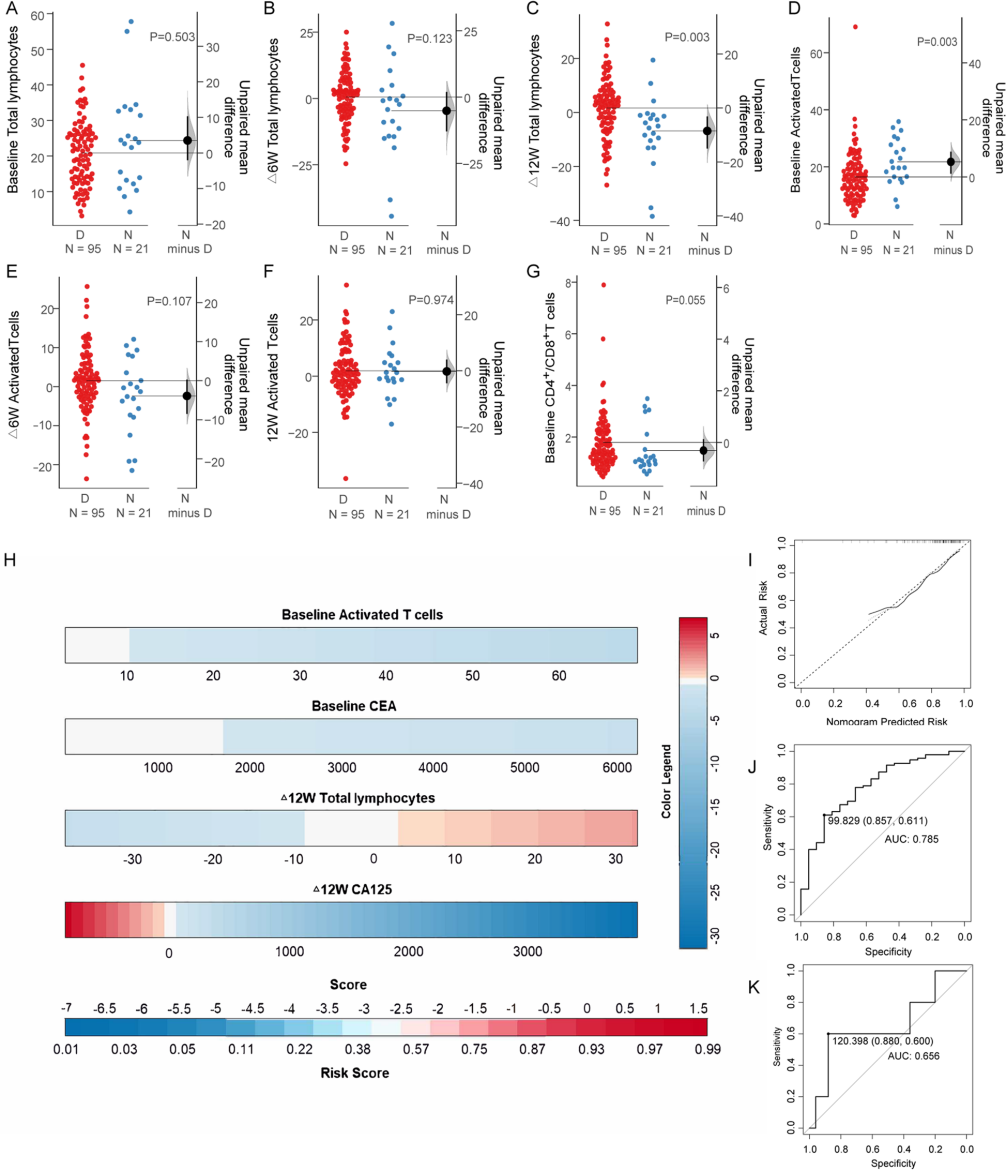
Analysis of therapeutic efficacy based on peripheral blood parameters. **A**-**G** Differences between DCB and NDB groups at baseline and post ICB treatment in the training, Nonparametric Mann-Whitney test was used for comparisons (using continuous variables). **H** Nomogram for predicting ICB treatment response of NSCLC patients. **I** Calibration curves of the nomogram. **J** ROC curves in the training set. **K** ROC curves in the validation set. DCB, Durable clinical benefit; NDB, None durable benefit; CEA, carcinoembryonic antigen; CA125, carbohydrate antigen 125; NSCLC, Non-small cell lung cancer; AUC, Area under the curve; ROC, receiver operating characteristic curve

**Fig. 3:**
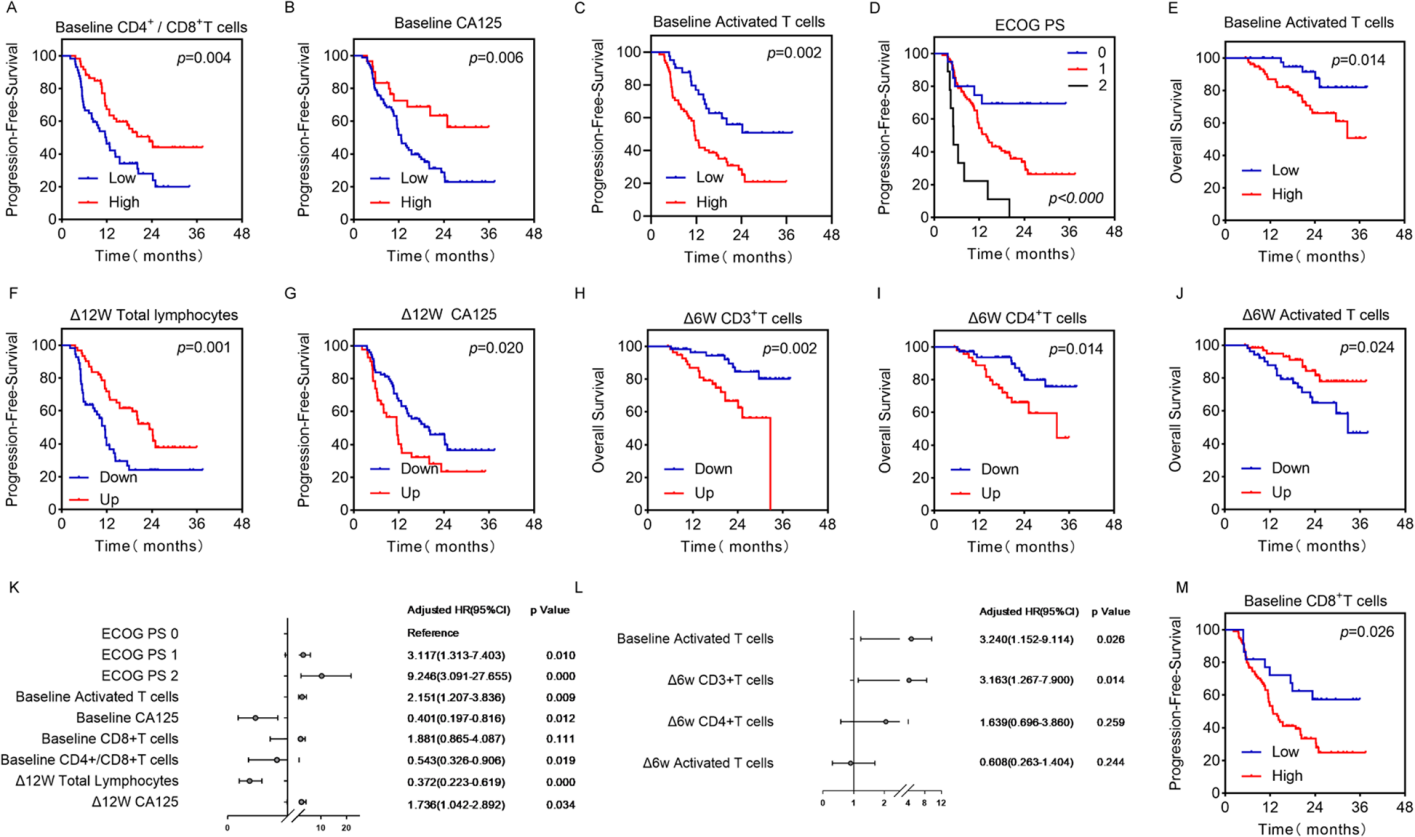
PFS and OS curves. **A**-**J** and **M** Kaplan–Meier curves for PFS or OS. **K** and **L** PFS and OS Hazard ratio and 95%CI show in the Forest plot, respectively. *P* Values were calculated by log-rank statistics. ECOG PS, Eastern Cooperative Oncology Group performance status; CA125, carbohydrate antigen 125. PFS, progression-free survival; OS, Overall survival

Before treatment, no total lymphocytes difference was found between DCB and NDB patients (Fig. 2A), after the administration of α-PD-1, the total lymphocytes in DCB patients gradually elevated, and a significant difference were observed at the week of 12 (Fig. 2B, C). Interestingly, we found that at baseline, lower activated T cell counts predict good ICB responses (but not in dynamic changes, Fig. 2D, E, F), but after treatment, the elevation of activated T cell counts predicts good outcomes (Fig. 3J). Other data in Supplementary Tables 7, Supplementary Fig. 2.

#### Nomogram model predicts immunotherapeutic responses

We constructed a nomogram to improve the predictive efficacy and clinical applicability (Fig. 2H). The calibration curve showed good correlation among the actual observations and estimates obtained (Fig. 2I). The AUC value and C-index of the nomogram model were both 0.785 (Fig. 2J). The AUC value and C-index of the validation set were both 0.656(Fig. 2K).

### Association between lymphocytes’ differentiation and patients’ outcomes

#### Baseline peripheral lymphocytes’ differentiation predicts immunotherapeutic outcomes

For disease progression (Supplementary Table 8), higher CD4^+^ / CD8^+^ T cell ratio and CA125 were significantly correlated with longer free progression time (Cox regression, *p* = 0.019, HR = 0.543, 95%CI:0.326–0.906; *p* = 0.012, HR = 0.401, 95%CI:0.197–0.816, respectively; Fig. 3A, B). In contrast, lower level of activated T cells was correlated with longer free progression time (*p* = 0.009, HR = 2.151, 95%CI:1.207–3.836; Fig. 3C). Better ECOG score predicts longer PFS time (1 vs. 0: *p* = 0.010, HR = 3.117, 95%CI:1.313–7.403; 2 vs. 0: *p* = 0.000, HR = 9.246, 95%CI:3.091–27.655; Fig. 3D). For the survival of the patient (Supplementary Table 9), activated T cell counts predict poor overall survival (*p* = 0.026, HR = 3.240, 95%CI:1.152–9.114; Fig. 3E).

#### Dynamic peripheral lymphocytes’ differentiation predicts immunotherapeutic outcomes

For evaluating the association between peripheral lymphocytes differentiation and patients’ outcomes, we introduced parameters with *p* value < 0.05 in univariable cox regression for multivariate analysis. We found that patients with increased total lymphocytes and decreased CA125 after 12 weeks of ICB treatment had longer PFS (mPFS: Up vs. Down: 23.3 m vs. 11.5 m, *p* = 0.000, HR = 0.372, 95%CI:0.223–0.619; mPFS: Up vs. Down: 11.5 m vs. 20 m *p* = 0.034, HR = 1.736, 95%CI:1.042–2.892, Fig. 3F, G, Supplementary Tables 8 and 10). After 6 weeks of treatment, total T, T_H_ and activated T cell counts correlate with longer OS (*p* = 0.003, *p* = 0.018, *p* = 0.029, respectively, Fig. 3H, I, J, Supplementary Table 9), and Δ6W total T cells were independent favorable prognostic factor for OS (mOS: Up vs. Down: 32.7 m vs. NR, *p* = 0.014, HR = 3.163, 95%CI:1.267-7.900, Supplementary Tables 9 and 10). Figure 3K, L shown the Hazard radios and 95% CI of the significant factors, and other data shown in Supplementary Tables 11 and 12, Supplementary Figs. 3–5.

#### Nomogram model predicts immunotherapeutic outcomes

**Fig. 4:**
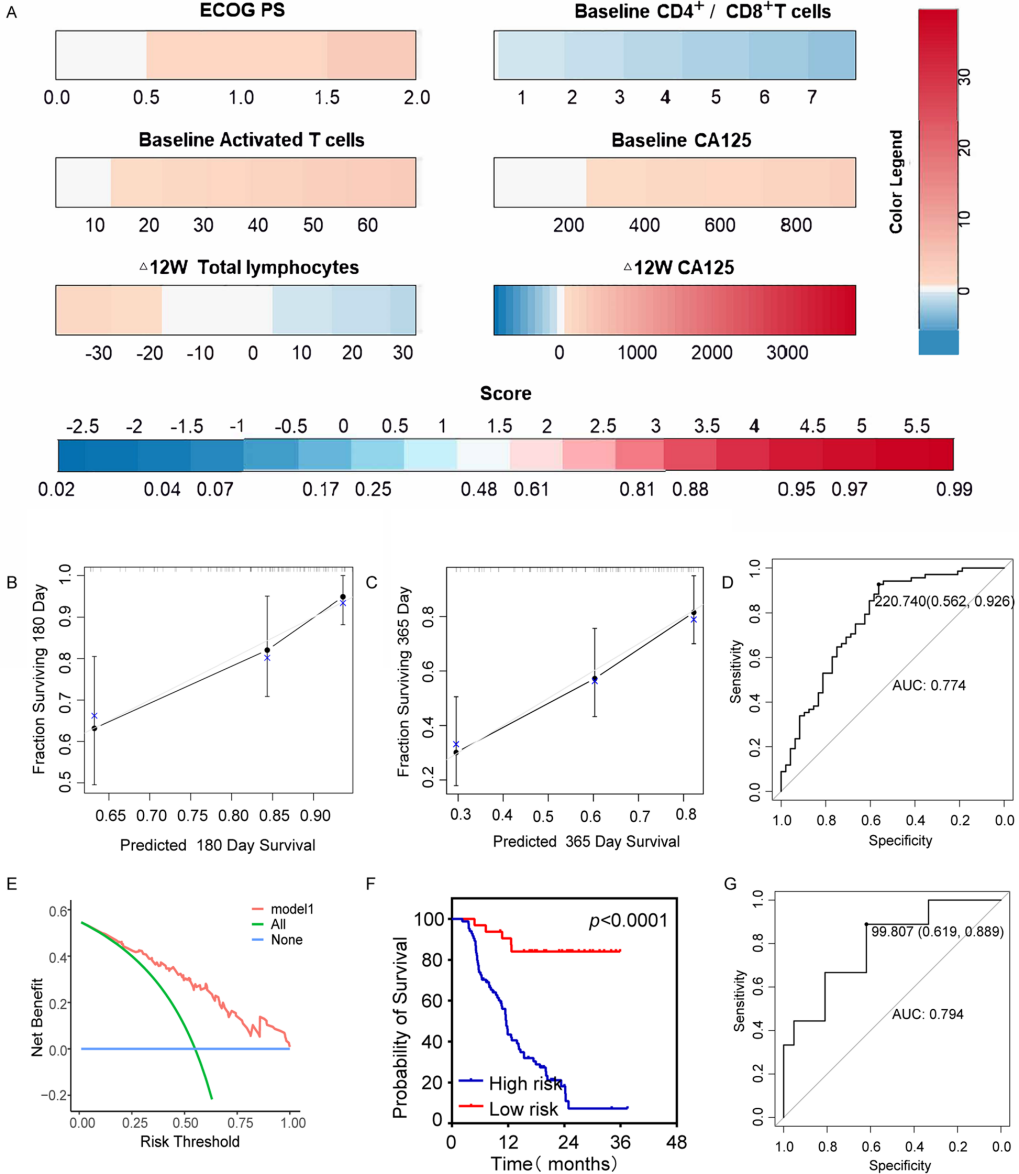
Nomogram model of disease progression. **A** PFS nomogram based on the multivariate model, including ECOG PS, Baseline Activated T cells, Baseline CD4+/CD8 + T cells, Baseline CA125, Δ12W Total lymphocytes andΔ12W CA125. **B **and **C** The 180- and 365- days PFS calibration curves. **D** ROC curves in the training set. **E** DCA of the nomogram. Model 1(DCA curves for PFS in the training set). **F** Kaplan-Meier curves of nomogram in training set. **G** ROC curves in the validation set. PFS, progression-free survival; ECOG PS, Eastern Cooperative Oncology Group performance status; DCA, decision curve analysis; ROC, receiver operating characteristic curve; AUC, area under the curve

For PFS prediction model (Fig. 4A), the 180- and 365-day calibration curves showed a good agreement between the actual and predicted outcomes (Fig. 4B, C). The AUC of training set was 0.774, and C-index was 0.728 (95%CI:0.696–0.760, Fig. 4D). The DCA curves for PFS in the training set was shown in Fig. 4E. Training set patients were categorized by risk score (Low / High risk), patients with lower risk scores had a longer PFS (HR = 0.113; 95% CI: 0.070–0.183; *P* < 0.0001; Fig. 4F). The AUC of validation set was 0.794, and C-index was 0.737 (95%CI:0.654–0.820, Fig. 4G).

**Fig. 5:**
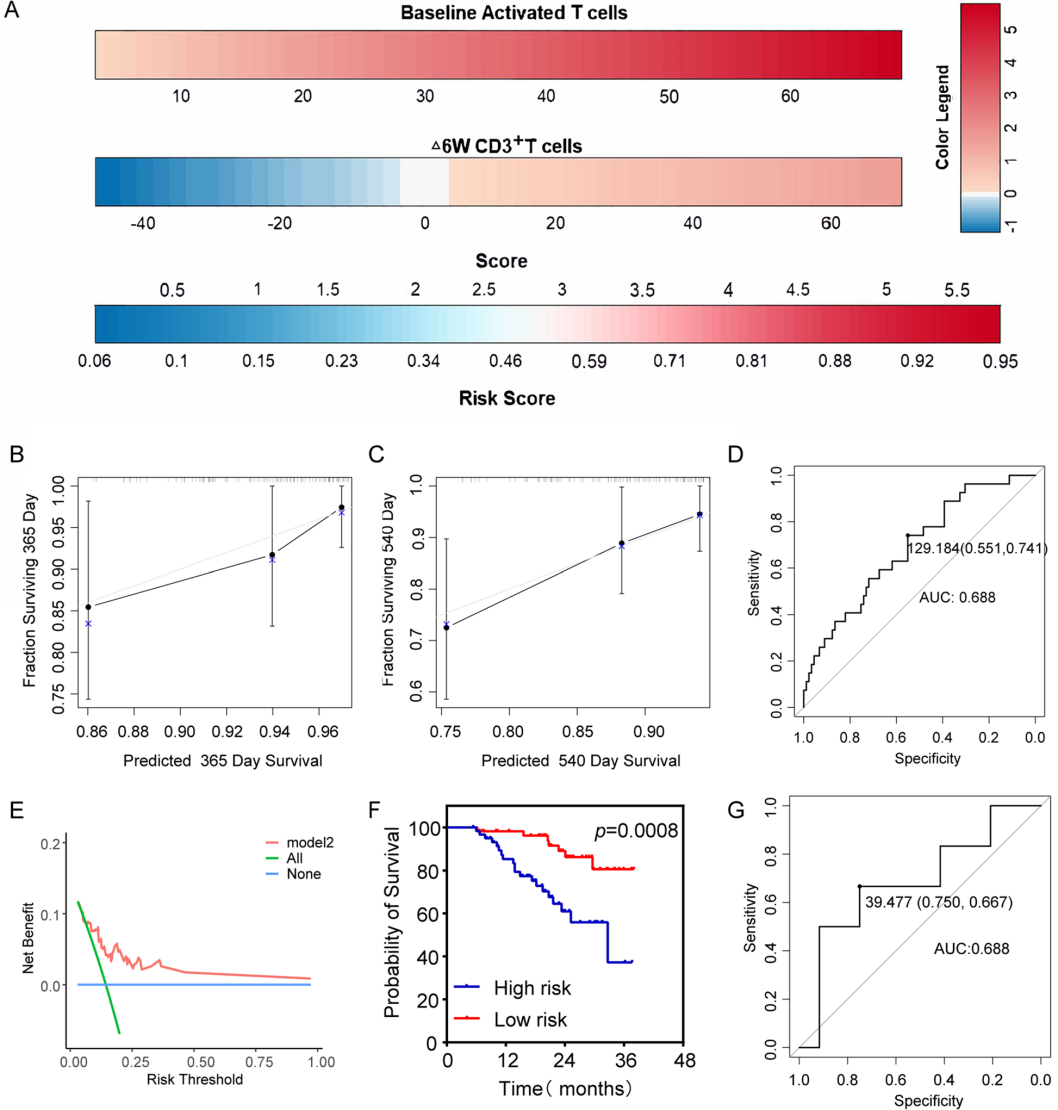
Nomogram model of overall survival. **A** OS nomogram based on the multivariate model, including Baseline Activated T cells, Δ6W CD3 + T cells. **B** and **C** The 365- and 540- days OS calibration curves. **D** ROC curves in the training set. **E** DCA of the nomogram. Model 2(DCA curves for OS in the training set). **F** Kaplan-Meier curves of nomogram in training set. **G** ROC curves in the validation set. OS, overall survival; DCA, decision curve analysis; ROC, receiver operating characteristic curve; AUC, area under the curve

For OS prediction model (Fig. 5A), the 365- and 540-day prediction curve of the model is close to the actual observation curve, showed the good calibration ability of the mode (Fig. 5B, C). The AUC of training set was 0.688, and C-index was 0.721 (95%CI:0.669–0.771, Fig. 5D). The DCA curves for OS in the training set is shown in Fig. 5E. Training set patients with lower risk scores had a longer OS (HR = 0.258; 95% CI: 0.120–0.553; *P* = 0.0008; Fig. 5F). The AUC of validation set was 0.688, and C-index was 0.639 (95%CI:0.0.531–0.747, Fig. 5G).

## Discussion

Lymphocytes which are differentiated from lymphoblasts-HSC (hematopoietic stem cells) circulate in peripheral blood and primary / secondary lymphoid organs and master adaptive immune responses / surveillance [27]. There are three major populations of lymphocytes, B, NK and T populations. And anti-tumor immunity is primarily conducted and regulated by several T subpopulations. In the thymus, T cells undergo positive and negative selection and differentiation into two major distinct subsets, CD4^+^ T_H_ / Regulatory T cells and CD8^+^ CTL cells [28]. Mature lymphocytes encounter antigens in secondary lymphoid organs and eventually differentiate into subpopulations of cells with different effector functions, such as activated T cells (HLA-DR^+^). And microenvironment PD-L1 (CD274) binds to PD-1 (CD279) which is mainly expressed on the surface of T cells and results in T cell exhausting (expressing CD39) [29], α-PD-1 monoclonal antibody blocks PD-1 on T cell surface, avoids CTL exhausting, facilitates cytokines releasing (e.g., Interferons-γ, which may also influence cell differentiation), and remodels lymphocytes differentiation / activation [30, 31].

In practice, classical biomarkers being examined before immunotherapy include TMB and PD-L1 [17, 32]. Several pieces of evidence suggested the failure of using these markers as biomarkers for ICB responses [19]. With the inadequacy of classical markers, more researchers focused on emerging biomarkers such as neoantigen patterns, gut microbiota, tertiary lymphoid structure, etc. [33, 34]. In the current study, we explored the predictive value of lymphocytes differentiation (baseline and dynamic changes) for ICB treatment responses / outcomes.

We analyzed the training set variables using machine learning and found that baseline activated T cells, Δ12W total lymphocytes, and baseline CD4+/CD8 + T cells were essential predictors of ICB prognosis. Subsequently, we constructed a clinical prediction model and validated it with a validation set, aiming at a comprehensive assessment of the variables.

Systemic immune dysregulation and cytotoxic agents induced hematopoietic damage together leading to lower peripheral lymphocytes in cancer patients [35], and people assume that low peripheral lymphocyte counts positively correlate with fewer tumor-infiltrating immunocytes and predict poor responses / outcomes [36]. Wang et al. reported that total lymphocyte count was higher in the ICB benefit group [37]. Different from previous reports indicating lymphocyte counts predict ICB responses / outcomes [38, 39], we didn’t find any statistical difference in total lymphocyte counts between DCB and NDB patients or survival / progression benefit between high and low lymphocyte counts at baseline (Fig. 2A, Supplementary Tables 11 and 12). For dynamic changes, previous studies reported the importance of increased lymphocytes after ICB treatment, we also found that increased lymphocyte counts after ICB treatment predicts good responses / outcomes at a week of 12 (Figs. 2C and 3F, Supplementary Tables 6 and 8) [37, 40]. In summary, we have showed the essential of monitoring lymphocyte counts during ICB treatment by flow cytometry.

As key players in immune surveillance and anti-tumor immunity, T cell activation featured with the expression of major histocompatibility complex class-II molecular (e.g., HLA-DR) requires both antigen-specific and costimulatory signals. And increased activated T cell (HLA-DR^+^) counts during ICB treatment indicate the success of ICB treatment [41, 42]. In current research, we also found that the elevation of activated T cells indicates better outcomes after a short period of treatment (6 weeks, Fig. 3J, Supplementary Table 9). But interestingly, at baseline, we found that higher activated T cells correlate with less clinical benefit (Fig. 2D, Supplementary Table 6), shorter PFS (Fig. 3C, Supplementary Table 8), or OS (Fig. 3E, Supplementary Table 9).

After that, we explored the distribution of T cells and their major subtypes, T_H_s and CTLs in peripheral blood. We found that patients with higher levels of CTLs count at baseline bear a poor prognosis (Fig. 3M, Supplementary Table 8), but an elevated CTLs level after 12 weeks of ICB treatment indicates favorable responses (Supplementary Table 5), our data complement previous knowledge indicating the importance of tumor-infiltrating CTLs level [43]. But interestingly, an increased total T cell counts was associated with poor responses / outcomes from a week of 6 (Fig. 3H, Supplementary Tables 5 and 9). This counterintuitive phenomenon might be explained by CD4^+^ / CD8^+^ T cell ratio, after 12 weeks of ICB treatment a decreased ratio was associated with favorable responses, considering in peripheral blood the majority of T cells are CD4^+^ T_H_s subpopulations which indicates the increased total T cell counts in NDB group might be explained by the elevation of T_H_s. And complement with previous data we also found that baseline CD4^+^ / CD8^+^ T cell ratio was positively associated with clinical benefits including responses and outcomes (Figs. 2G and 3A) [44, 45].

Tumor markers were commonly used as auxiliary biomarkers for cancer diagnosis. Currently, CA125 is mainly considered as a specific tumor marker for ovarian cancer, but several studies showed that CA125 was elevated in about 46.6% of NSCLC patients, and predicts worse outcomes / aggressive phenotypes [46, 47]. In the current study, we found that patients with higher level of CA125 at baseline have better outcomes, but an elevated CA125 level after 12 weeks of ICB treatment indicates worse ICB response and outcomes (Fig. 3B, G Supplementary Tables 6 and 8).

We constructed clinical prediction models for ICB treatment response and outcome in the training set, and validated the model with the validation set (Figs. 3, 4 and 5). Our model showed moderate prediction performance for immunotherapeutic responses and outcomes, and it can provide intuitive initial treatment expectation for clinicians.

Some limitations should be addressed for current research. Firstly, because PD-L1 immunohistochemistry staining is not a mandatory test for patients who will receive 2+-line therapy or in combination with platinum based first-line therapy, therefore no PD-L1 tumor proportion score (PD-L1 TPS) expression was recorded and reported in current study. Secondly, the patients enrolled in this study were treated in different clinical groups from our hospital, it’s difficult to fully record the immune-related adverse events (irAE). Thirdly, the retrospect study with fewer markers for flow cytometry panel limited the exploration of immunophenotype, a prospective study with more flow cytometry makers is required for fully understanding the relationship between ICB outcomes / responses and immunophenotypes.

In the current study, we focused on analyzing the dynamic changes of peripheral blood lymphocytes differentiation characteristics in patients receiving ICB treatment. We observed distinctive modification of immune status in certain groups of patients with favorable responses / outcomes after immunotherapy (e.g., elevated activated T cell counts after ICB treatment), which might help to select and identify novel therapeutic beneficiaries. Moreover, precise identification of more subpopulations using other lymphocyte markers might provide richer results, and further studies using larger cohorts of patients with control arms are warranted to validate these biomarkers.

## Supplementary Information

**Additional file 1:** **Supplement Fig. 1. **Process of research and experimental gating strategy**.** (A) Diagram of peripheral blood sample collection, treatment and treatment efficacy evaluation. (B) Flowchart of study design. (C) Flow cytometry gating strategy. Leukocytes were first identified based on CD45 expression. Lymphocytes were identified based on forward (size) and side (granularity) scatted characteristics. Expression of CD3 identified Total T cells (CD3^+^), and were further subdivided based on CD4 and CD8 expression into T_H_(CD3^+^CD4^+^) and CTL(CD3^+^CD8^+^) cells. Expression of HLA-DR and CD56 identified the Activated T cells (HLADR^+^) and NKT cells (CD56^+^). Expression of CD19 / CD56 but do not express CD3 were identified as B cells (CD3^-^CD19^+^) and NK cells (CD3^-^CD56^+^). **Supplement Fig. 2.** Differences between DCB and NDB groups at baseline and post ICB treatment in the training. Nonparametric Mann-Whitney test was used for comparisons (using continuous variables). Durable clinical benefit; NDB, None durable benefit; NK, natural killer; NKT, natural killer T; CEA, carcinoembryonic antigen; CA125, carbohydrate antigen 125; CA199, carbohydrate antigen 199; NSE, neuron-specific enolase. **Supplement Fig. 3. **Overall population Kaplan–Meier curves for PFS(A) and OS(B). DCB and NDB group Kaplan–Meier curves for (C)PFS and (D)OS. *P* Values were calculated by log-rank statistics. Durable clinical benefit; NDB, None durable benefit; PFS, progression-free survival; OS, overall survival. **Supplement Fig. 4. **Kaplan–Meier curves forPFS. *P* Values were calculated by log-rank statistics. **Supplement Fig. 5. **Kaplan–Meier curves for OS. *P* Values were calculated by log-rank statistics. **Supplement Fig. 6. **Relationship between infiltrating lymphocytes and prognosis in NSCLC patients with TCGA. Whole-transcriptome RNA-seq data for 337 NSCLC cases and their corresponding clinical data were downloaded from the TCGA database (https://portal.gdc.cancer.gov/). Immuno-infiltrating cells were calculated with the xCell R package. Patients’ parameters were categorized by optimal cut-off values (Low / High group), The survival curve was plotted using the Kaplan–Meier method and the log rank test was used to determine statistical significance; *p*<0.05 was considered statistically significant. The Cancer Genome Atlas Program, TCGA; Central memory T cells, Tcm; Effector memory T Cells, Tem; Helper T cell, TH. **Supplementary Table 1. **Clinical characteristics of Training set and Validation set. **Supplementary Table 2. **Flow cytometry antibody list. **Supplementary Table 3. **Training set baseline peripheral blood parameters. **Supplementary Table 4. **Training set patients’ peripheral blood parameters at Δ6W, Δ12W. **Supplementary Table 5. **The association between patient peripheral blood parameters and treatment response. **Supplementary Table 6.** Multivariable Logistic regression models for DCB. **Supplementary Table 7.** Multivariable Logistic regression of DCB. **Supplementary Table 8.** Univariable and Multivariable Cox regression analysis of Progression free survival. **Supplementary Table 9.** Univariable and Multivariable Cox regression analysis of Overall survival. **Supplementary Table 10. **Median months of PFS and OS in each group. **Supplementary Table 11. **Univariable Cox regression of Progression free survival. **Supplementary Table 12. **Univariable Cox regression of Overall survival.

## Data Availability

All the data generated in this study are included in the article.

## Abbreviations

PD-1: Programmed cell death protein 1
PD-L1: Programmed cell death-Ligand 1
NSCLC: Non-small cell lung cancer
ICB: Immune checkpoint blockade
FCM: Flow cytometry
TMB: Tumor mutation burden
CEA: Carcinoembryonic antigen
CA199: Carbohydrate antigen 199
CA125: Carbohydrate antigen 125
NSE: Neuron-specific enolase
CR: Complete response
PR: Partial response
SD: Stable disease
PD: Progressed disease
DCB: Durable clinical benefit
NDB: None durable benefit
PFS: Progression-free survival
OS: Overall survival
PBMC: Peripheral blood mononuclear cells
NK: Natural killer cell
NKT: Natural killer T cell
LASSO: Least absolute shrinkage and selection operator
RF: Random Forest
XGBoost: Extreme gradient boosting
AUC: Area under the curve
DCA: Decision curve analysis
ECOG PS: Eastern Cooperative Oncology Group performance status
ROC: Rreceiver operating characteristic curve
CTL: Cytotoxic T cell
TPS: Tumor proportion score
irAE: Immune-related adverse events
TCGA: The Cancer Genome Atlas Program
Tcm: Central memory T cell
Tem: Effector memory T cell
TH: Helper T cell
