# Supplementary material for: Peripheral blood lymphocytes differentiation patterns in responses / outcomes to immune checkpoint blockade therapies in non-small cell lung cancer: a retrospective study
Source: BMC Cancer. 2023 Jan 25;23:83. doi: 10.1186/s12885-023-10502-4 (PMC9875514; doi:10.1186/s12885-023-10502-4)
Supplement: Supplementary file 1 — Additional file 1: Supplement Fig. 1. Process of research and experimental gating strategy. (A) Diagram of peripheral blood sample collection, treatment and treatment efficacy evaluation. (B) Flowchart of study design. (C) Flow cytometry gating strategy. Leukocytes were first identified based on CD45 expression. Lymphocytes were identified based on forward (size) and side (granularity) scatted characteristics. Expression of CD3 identified Total T cells (CD3+), and were further subdivided based on CD4 and CD8 expression into TH(CD3+CD4+) and CTL(CD3+CD8+) cells. Expression of HLA-DR and CD56 identified the Activated T cells (HLADR+) and NKT cells (CD56+). Expression of CD19 / CD56 but do not express CD3 were identified as B cells (CD3-CD19+) and NK cells (CD3-CD56+). Supplement Fig. 2. Differences between DCB and NDB groups at baseline and post ICB treatment in the training. Nonparametric Mann-Whitney test was used for comparisons (using continuous variables). Durable clinical benefit; NDB, None durable benefit; NK, natural killer; NKT, natural killer T; CEA, carcinoembryonic antigen; CA125, carbohydrate antigen 125; CA199, carbohydrate antigen 199; NSE, neuron-specific enolase. Supplement Fig. 3. Overall population Kaplan–Meier curves for PFS(A) and OS(B). DCB and NDB group Kaplan–Meier curves for (C)PFS and (D)OS. P Values were calculated by log-rank statistics. Durable clinical benefit; NDB, None durable benefit; PFS, progression-free survival; OS, overall survival. Supplement Fig. 4. Kaplan–Meier curves forPFS. P Values were calculated by log-rank statistics. Supplement Fig. 5. Kaplan–Meier curves for OS. P Values were calculated by log-rank statistics. Supplement Fig. 6. Relationship between infiltrating lymphocytes and prognosis in NSCLC patients with TCGA. Whole-transcriptome RNA-seq data for 337 NSCLC cases and their corresponding clinical data were downloaded from the TCGA database (https://portal.gdc.cancer.gov/). Immuno-infiltrating cells were calculated w [file 12885_2023_10502_MOESM1_ESM.docx]

**Peripheral blood lymphocytes differentiation patterns in responses / outcomes to immune checkpoint blockade therapies in non-small cell lung cancer: a retrospective study**

**
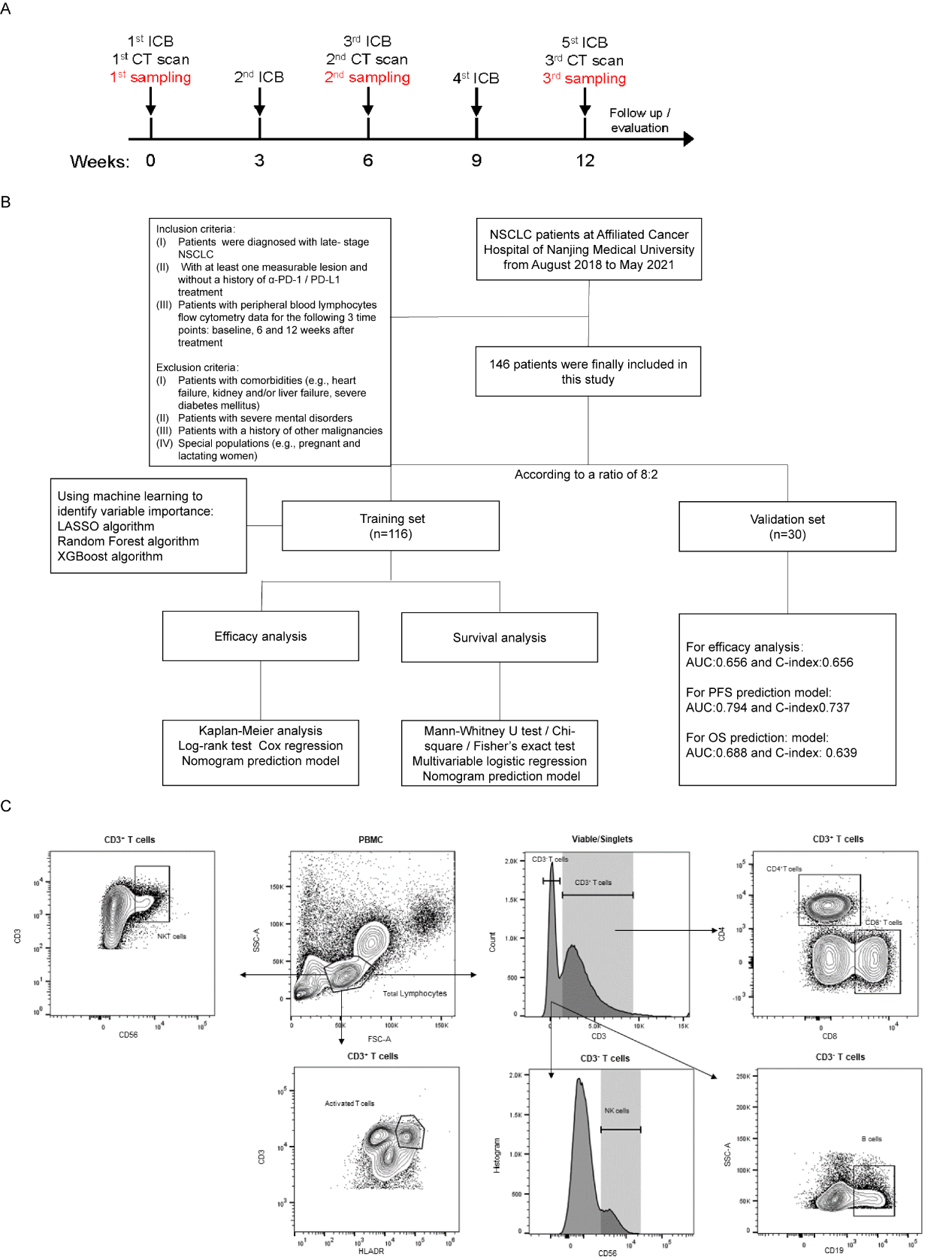
**

**Supplement Fig.1** Process of research and experimental gating strategy**.** (A) Diagram of peripheral blood sample collection, treatment and treatment efficacy evaluation. (B) Flowchart of study design. (C) Flow cytometry gating strategy. Leukocytes were first identified based on CD45 expression. Lymphocytes were identified based on forward (size) and side (granularity) scatted characteristics. Expression of CD3 identified Total T cells (CD3^+^), and were further subdivided based on CD4 and CD8 expression into T_H_(CD3^+^CD4^+^) and CTL(CD3^+^CD8^+^) cells. Expression of HLA-DR and CD56 identified the Activated T cells (HLADR^+^) and NKT cells (CD56^+^). Expression of CD19 / CD56 but do not express CD3 were identified as B cells (CD3^-^CD19^+^) and NK cells (CD3^-^CD56^+^).


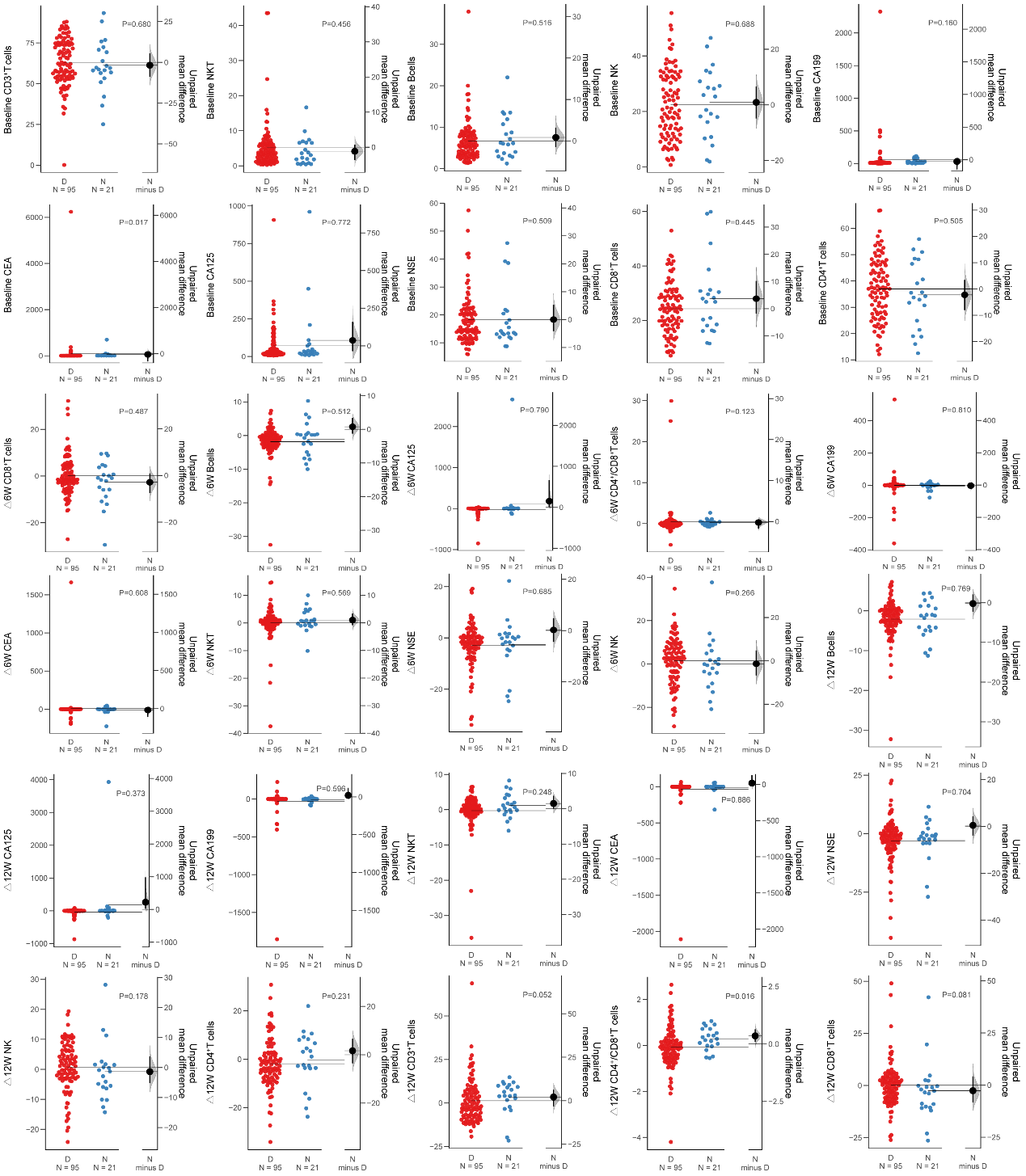


**Supplement Fig.2** Differences between DCB and NDB groups at baseline and post ICB treatment in the training. Nonparametric Mann-Whitney test was used for comparisons (using continuous variables). Durable clinical benefit; NDB, None durable benefit; NK, natural killer; NKT, natural killer T; CEA, carcinoembryonic antigen; CA125, carbohydrate antigen 125; CA199, carbohydrate antigen 199; NSE, neuron-specific enolase.


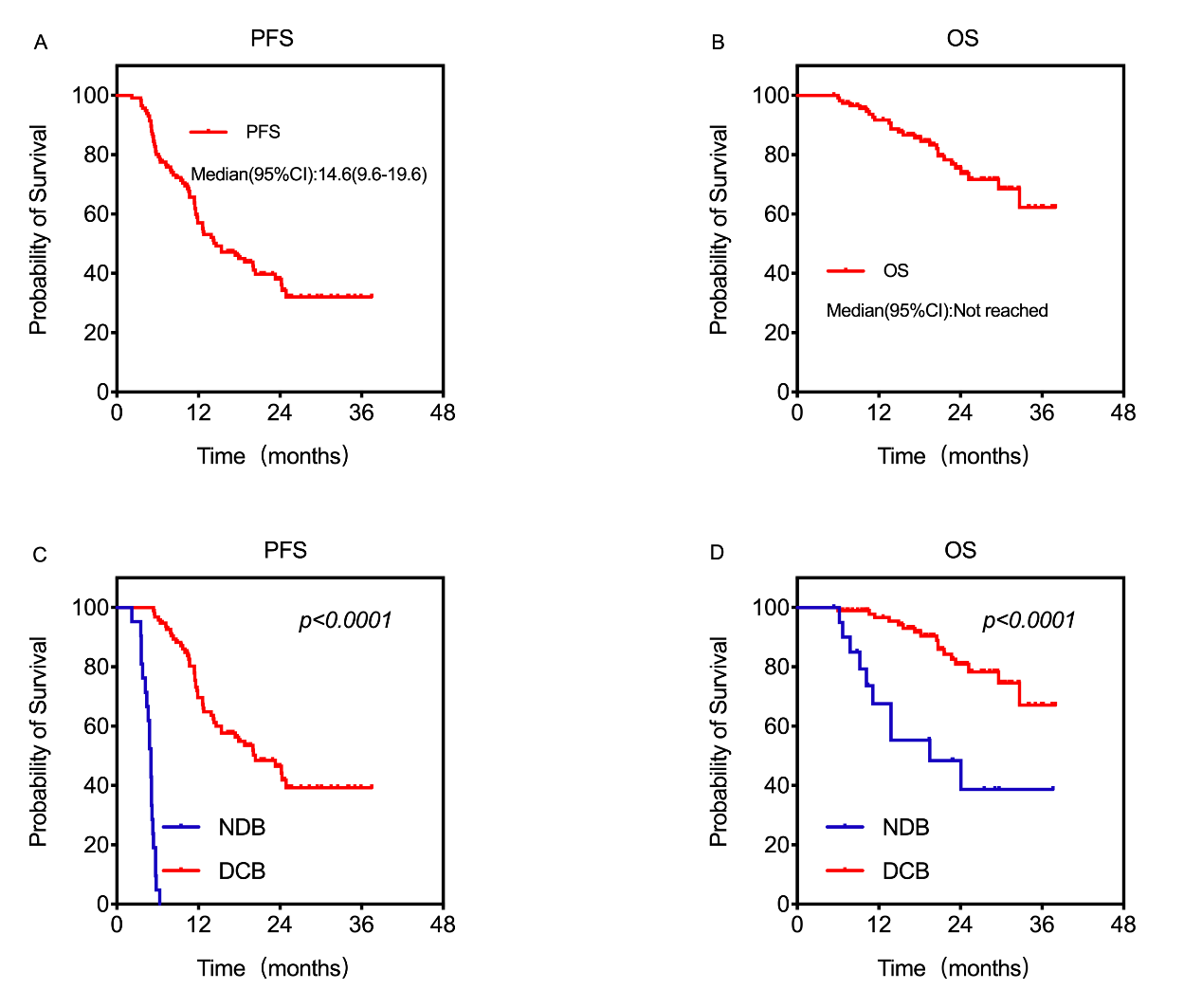


**Supplement Fig.3** Overall population Kaplan–Meier curves for PFS(A) and OS(B). DCB and NDB group Kaplan–Meier curves for (C)PFS and (D)OS. P Values were calculated by log-rank statistics. Durable clinical benefit; NDB, None durable benefit; PFS, progression-free survival; OS, overall survival.


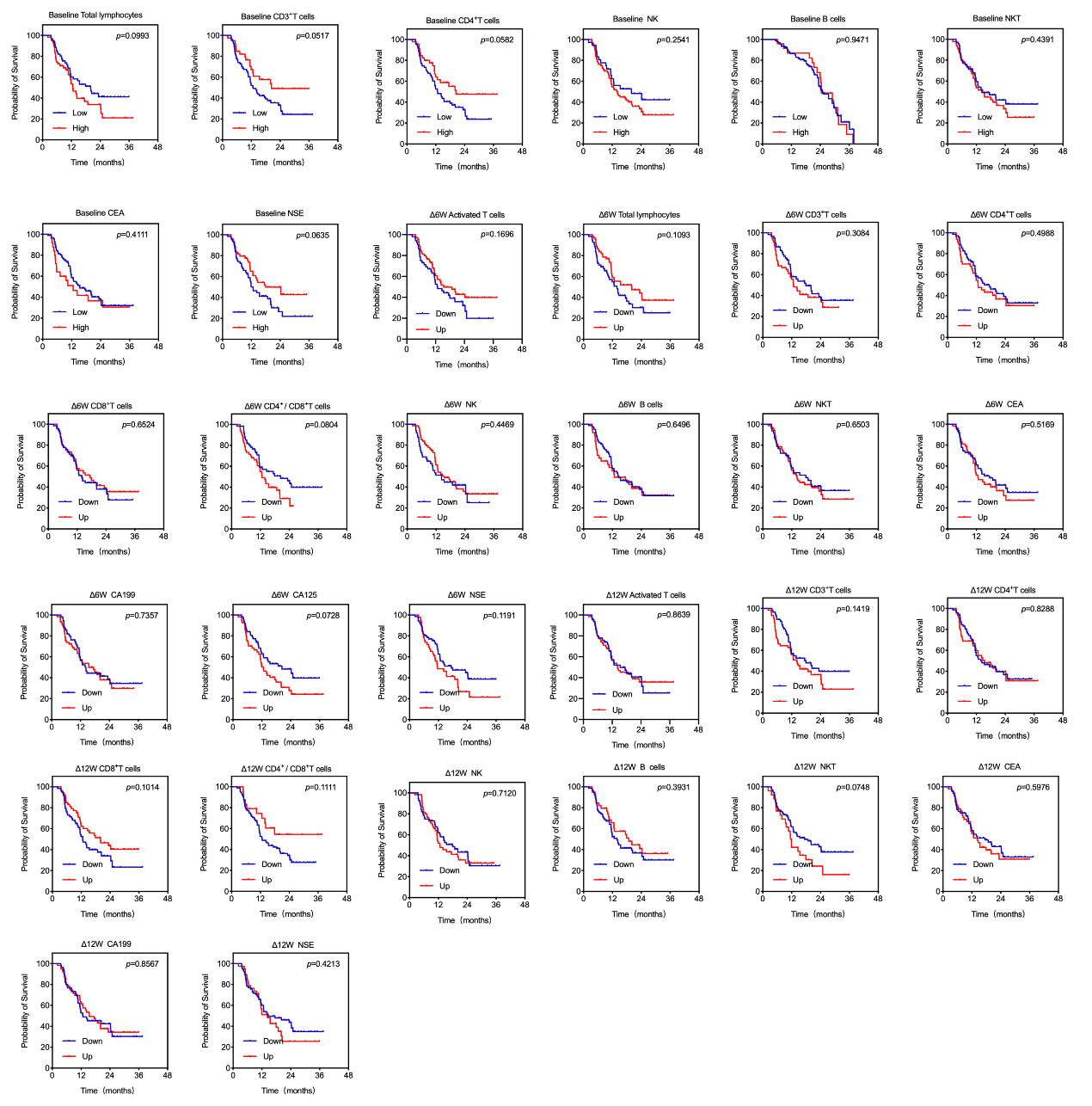


**Supplement Fig.4** Kaplan–Meier curves for PFS. P Values were calculated by log-rank statistics.

**
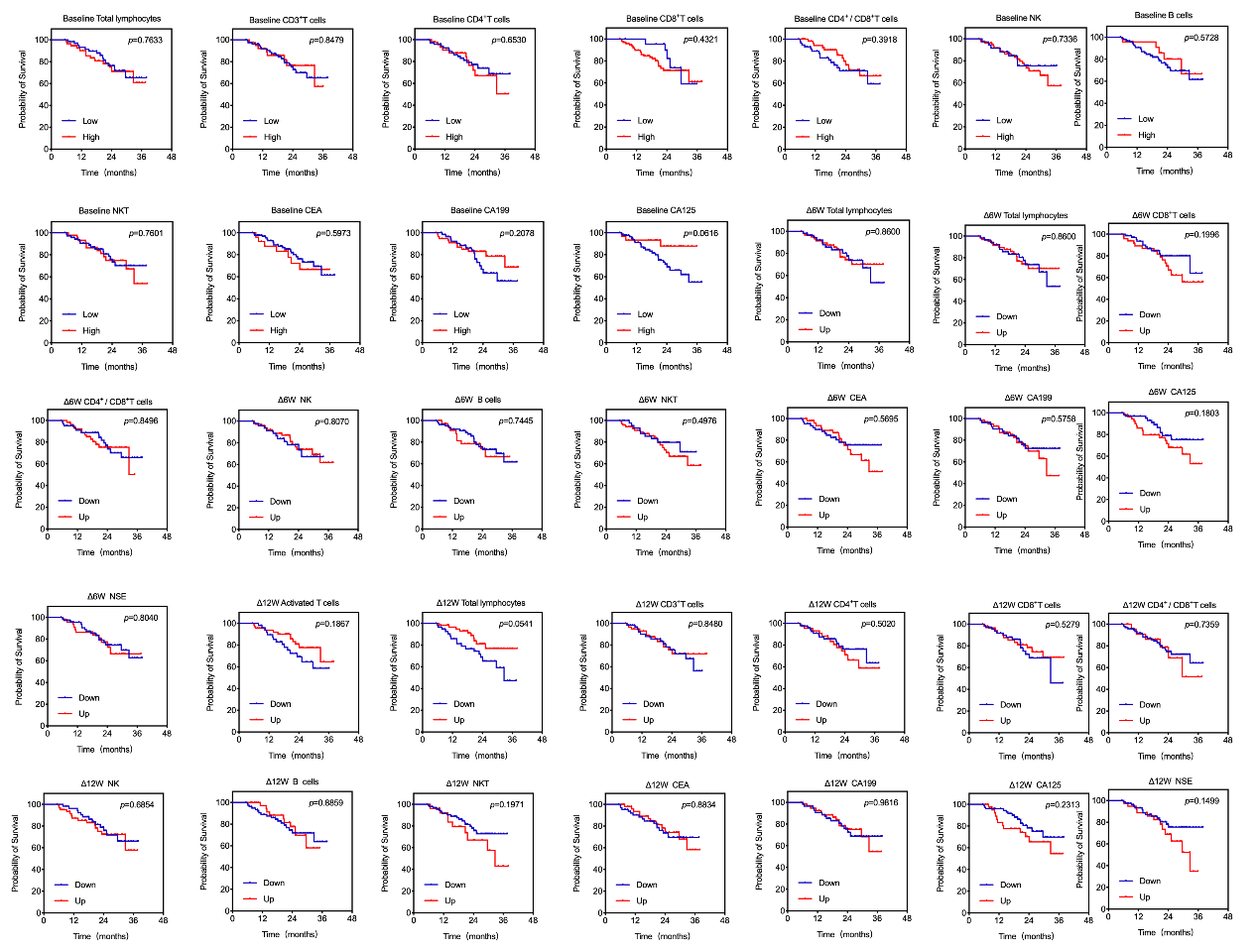
**

**Supplement Fig.5** Kaplan–Meier curves for OS. P Values were calculated by log-rank statistics.


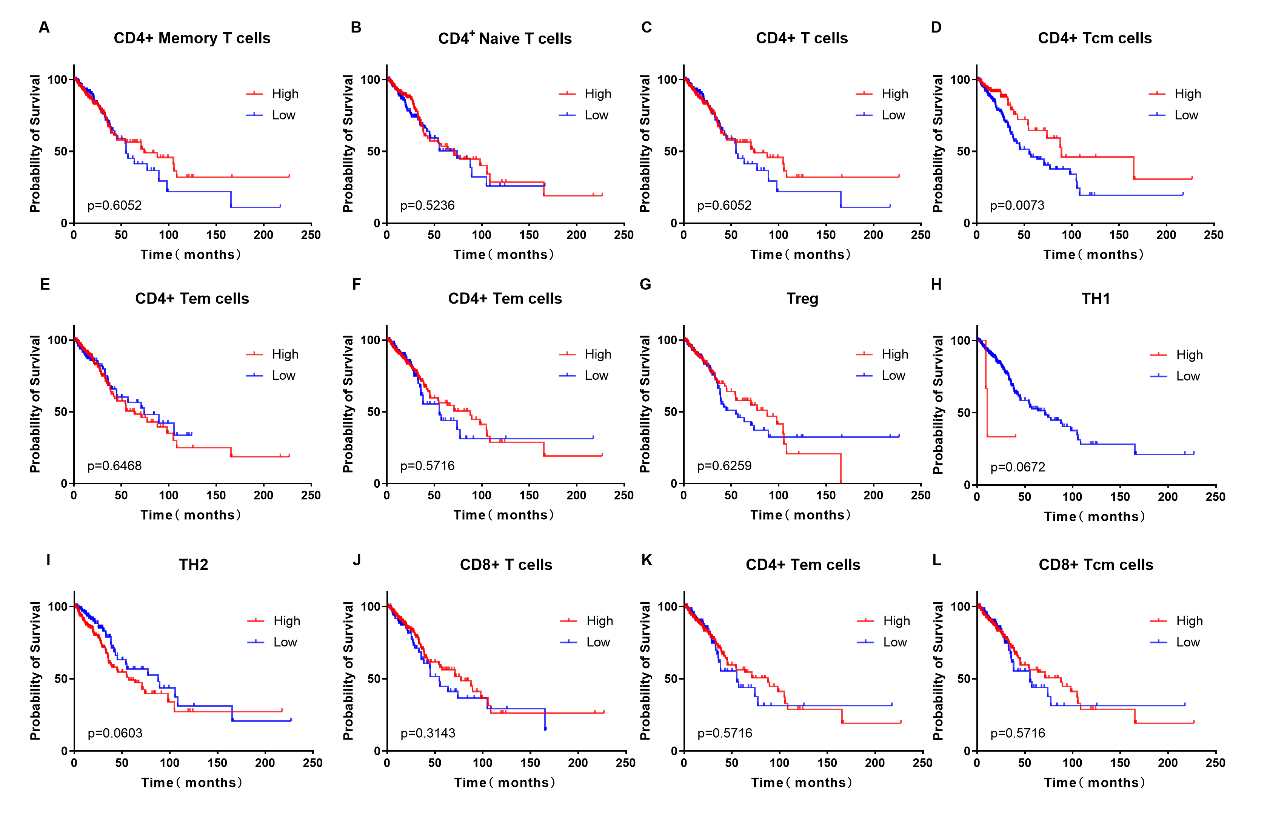


**Supplement Fig.6** Relationship between infiltrating lymphocytes and prognosis in NSCLC patients with TCGA. Whole-transcriptome RNA-seq data for 337 NSCLC cases and their corresponding clinical data were downloaded from the TCGA database(<https://portal.gdc.cancer.gov/>). Immuno-infiltrating cells were calculated with the xCell R package. Patients’ parameters were categorized by optimal cut-off values (Low / High group), The survival curve was plotted using the Kaplan–Meier method and the log rank test was used to determine statistical significance; p<0.05 was considered statistically significant. The Cancer Genome Atlas Program, TCGA; Central memory T cells, Tcm; Effector memory T Cells, Tem; Helper T cell, TH.

**Supplementary Table1** Clinical characteristics of Training set and Validation set

|  | Total  (N=146) | Training set  (N=116) | Validation set  (N=30) |
| --- | --- | --- | --- |
| Age(years) ^a^ | 64(55-69) |  |  |
| <64 | 72 (49.3) | 59 (50.9) | 13 (43.3) |
| ≥64 | 74 (50.7) | 57 (49.1) | 17 (56.7) |
| Gender ^b^ |  |  |  |
| Male | 111 (76.0) | 89 (76.7) | 22 (73.3) |
| Female | 35 (24.0) | 27 (23.3) | 8 (26.7) |
| Histology ^b^ |  |  |  |
| Non- Squamous | 101 (69.2) | 79 (68.1) | 22 (73.3) |
| Squamous | 45 (30.8) | 37 (73.3) | 8 (26.7) |
| Stage ^b^ |  |  |  |
| IIIB-C | 27 (18.5) | 28 (15.5) | 9 (30.0) |
| IV | 119 (81.5) | 98 (84.5) | 21 (70.0) |
| Differentiation ^b^ |  |  |  |
| Moderate | 13 (8.9) | 7 (6.0) | 6 (20.0) |
| Medium-Low | 18 (12.3) | 16 (13.8) | 2 (6.7) |
| Low | 34 (23.3) | 29 (25.0) | 5 (16.7) |
| NA | 81 (55.5) | 64 (55.2) | 17 (56.7) |
| ECOG PS ^b^ |  |  |  |
| 0 | 23 (15.8) | 20 (17.2) | 3 (10.0) |
| 1 | 108 (74.0) | 87 (75.0) | 21 (70.0) |
| 2 | 15 (10.3) | 9 (7.8) | 6 (20.0) |
| Smoking history ^b^ |  |  |  |
| Never | 62 (42.5) | 43 (37.1) | 19 (63.3) |
| Now/Ever | 84 (57.5) | 73 (62.9) | 11 (36.7) |
| Distant metastases ^b^ |  |  |  |
| No | 27 (18.5) | 20 (17.2) | 9 (30.0) |
| Yes | 119 (81.5) | 96 (82.8) | 21 (70.0) |
| Driver mutations ^b^ |  |  |  |
| No | 113 (77.4) | 88 (75.9) | 25 (83.3) |
| Yes | 33 (22.6) | 28 (24.1) | 5 (16.7) |
| PD-1 inhibitor type ^b^ |  |  |  |
| Pembrolizumab | 47 (32.2) | 45 (38.8) | 2 (6.7) |
| Toripalimab | 18 (12.3) | 15 (12.9) | 3 (10.0) |
| Camrelizumab | 31 (21.2) | 20 (17.2) | 11 (36.7) |
| Sintilimab | 44 (30.1) | 36 (31.0) | 8 (26.7) |
| Tislelizumab | 6 (4.1) | 0 (0.0) | 6 (20.0) |
| Combination regimen ^b^ |  |  |  |
| Monotherapy | 25 (17.1) | 25 (21.6) | 0 (0.0) |
| Chemotherapy | 89 (61.0) | 64 (55.2) | 25 (83.3) |
| Anti-angiogenic therapy | 28 (19.2) | 27 (23.3) | 1 (3.3) |
| Both | 4 (2.7) | 0 (0.0) | 4 (13.3) |
| Drug regiment ^b^ |  |  |  |
| 1^st^ line | 62 (42.5) | 51 (44.0) | 11 (36.7) |
| ≥2^nd^ line | 84 (57.5) | 65 (56.0) | 19 (63.3) |
| Radiotherapy ^b^ |  |  |  |
| No | 69 (47.3) | 53 (45.7) | 16 (53.3) |
| Yes | 77 (52.7) | 63 (54.3) | 14 (46.7) |

^a^ Median and interquartile range (IQR); ^b^ N (%). ECOG PS, Eastern Cooperative Oncology Group Performance Status; PD-1, Programmed cell death protein 1; CR, Complete response; PR, Partial response; SD, Stable disease; PD, Progressed disease.

**Supplementary Table2** Flow cytometry antibody list

| Antigen | Fluorochrome | Source |
| --- | --- | --- |
| CD45 | PerCP-cy5.5 | BD |
| CD3 | FITC / APC | BD |
| CD4 | PE-cy7 | BD |
| CD8 | APC-cy7 | BD |
| CD56 | PE | BD |
| CD19 | APC | BD |
| HLA-DR | FITC | BD |

**Supplementary Table3** Training set baseline peripheral blood parameters

| Parameters | No. of patients (N=116) | Percentage (%) |
| --- | --- | --- |
| Baseline Total lymphocytes, median ^a^ | 21.83 (13.52-26.91) |  |
| <22.258 | 61 | 52.6 |
| ≥22.258 | 55 | 47.4 |
| Baseline CD3^+^T cells, median ^a^ | 61.70 (53.83-74.25) |  |
| <70.950 | 77 | 66.4 |
| ≥70.950 | 39 | 33.6 |
| Baseline CD4^+^T cells, median ^a^ | 36.50 (27.43-46.20) |  |
| <39.950 | 71 | 61.2 |
| ≥39.950 | 45 | 38.8 |
| Baseline CD8^+^T cells, median ^a^ | 24.35 (18.10-30.38) |  |
| <16.050 | 22 | 19.0 |
| ≥16.050 | 94 | 81.0 |
| Baseline CD4^+^ / CD8^+^T cells, median ^a^ | 1.42 (1.06-2.22) |  |
| <1.400 | 57 | 49.1 |
| ≥1.400 | 59 | 50.9 |
| Baseline NK, median ^a^ | 20.90 (11.81-32.56) |  |
| <14.304 | 36 | 31.0 |
| ≥14.304 | 80 | 69.0 |
| Baseline B cells, median ^a^ | 5.80 (3.43-8.48) |  |
| <8.800 | 92 | 79.3 |
| ≥8.800 | 24 | 20.7 |
| Baseline NKT, median ^a^ | 3.62 (1.69-6.38) |  |
| <4.569 | 70 | 60.3 |
| ≥4.569 | 46 | 39.7 |
| Baseline Activated T cells, median ^a^ | 16.53 (11.69-21.78) |  |
| <13.450 | 41 | 35.3 |
| ≥13.450 | 75 | 64.7 |
| Baseline CEA, median ^a^ | 4.88 (2.63-21.31) |  |
| <29.850 | 91 | 78.4 |
| ≥29.850 | 25 | 21.6 |
| Baseline CA125, median ^a^ | 28.83 (16.10-78.58) |  |
| <74.215 | 86 | 74.1 |
| ≥74.215 | 30 | 25.9 |
| Baseline CA199, median ^a^ | 13.37 (8.77-24.85) |  |
| <14.000 | 59 | 50.9 |
| ≥14.000 | 57 | 49.1 |
| Baseline NSE, median ^a^ | 15.24 (12.26-21.32) |  |
| <16.470 | 62 | 53.4 |
| ≥16.470 | 54 | 46.6 |

^a^ Median and interquartile range (IQR); NK, natural killer; NKT, natural killer T; CEA, carcinoembryonic antigen; CA125, carbohydrate antigen 125; CA199, carbohydrate antigen199; NSE**, neuron-specific enolase.**

**Supplementary Table4** Training set patients’ peripheral blood parameters at Δ6W, Δ12W

| Parameters | No. of patients (N=116) | Percentage (%) |
| --- | --- | --- |
| Δ6W Total lymphocytes |  |  |
| Up / Down | 61 / 55 | 52.6 / 47.4 |
| Δ6W CD3^+^T cells |  |  |
| Up / Down | 57 / 59 | 49.1 / 50.9 |
| Δ6W CD4^+^T cells |  |  |
| Up / Down | 47 / 69 | 40.5 / 59.5 |
| Δ6W CD8^+^T cells |  |  |
| Up / Down | 49 / 67 | 42.2 / 57.8 |
| Δ6W CD4^+^ / CD8^+^T cells |  |  |
| Up / Down | 56 / 60 | 48.3 / 51.7 |
| Δ6W NK |  |  |
| Up / Down | 68 / 48 | 58.6 / 41.4 |
| Δ6W B cells |  |  |
| Up / Down | 37 / 79 | 31.9 / 68.1 |
| Δ6W NKT |  |  |
| Up / Down | 69 / 47 | 59.5 / 40.5 |
| Δ6W Activated T cells |  |  |
| Up / Down | 64 / 52 | 55.2 / 44.8 |
| Δ6W CEA |  |  |
| Up / Down | 53 / 63 | 45.7 / 54.3 |
| Δ6W CA125 |  |  |
| Up / Down | 54 / 62 | 46.6 / 53.4 |
| Δ6W CA199 |  |  |
| Up / Down | 61 / 55 | 52.6 / 47.4 |
| Δ6W NSE |  |  |
| Up / Down | 46 / 70 | 39.7 / 60.3 |
| Δ12W Total lymphocytes |  |  |
| Up / Down | 61 / 55 | 52.6 / 47.4 |
| Δ12W CD3^+^T cells |  |  |
| Up / Down | 59 / 57 | 50.9 / 49.1 |
| Δ12W CD4^+^T cells |  |  |
| Up / Down | 45 / 71 | 38.8 / 61.2 |
| Δ12W CD8^+^T cells |  |  |
| Up / Down | 53 / 63 | 45.7 / 54.3 |
| Δ12W CD4^+^ / CD8^+^T cells |  |  |
| Up / Down | 57 / 59 | 49.1 / 50.9 |
| Δ12W NK |  |  |
| Up / Down | 60 / 56 | 51.7 / 48.3 |
| Δ12W B cells |  |  |
| Up / Down | 39 / 77 | 33.6 / 66.4 |
| Δ12W NKT |  |  |
| Up / Down | 65 / 51 | 56.0 / 44.0 |
| Δ12W Activated T cells |  |  |
| Up / Down | 66 / 50 | 56.9 / 43.1 |
| Δ12W CEA |  |  |
| Up / Down | 52 / 64 | 44.8 / 55.2 |
| Δ12W CA125 |  |  |
| Up / Down | 42 / 74 | 36.2 / 63.8 |
| Δ12W CA199 |  |  |
| Up / Down | 60 / 56 | 51.7 / 48.3 |
| Δ12W NSE |  |  |
| Up / Down | 38 / 78 | 32.8 / 67.2 |

NK, natural killer; NKT, natural killer T; CEA, carcinoembryonic antigen; CA125, carbohydrate antigen 125; CA199, carbohydrate antigen199; NSE, neuron-specific enolase.

**Supplementary Table5** The association between patient peripheral blood parameters and treatment response

| Parameters | Durable clinical benefit | | |
| --- | --- | --- | --- |
|  | N (%) | χ^2^ | P value |
| Age |  |  |  |
| <63 | 40/52 (42.1) |  |  |
| ≥63 | 55/64 (57.9) | 1.572 | 0.210 |
| Gender |  |  |  |
| Female | 22/27 (23.2) |  |  |
| Male | 73/89 (76.8) | 0.004 | 0.949 |
| Histology |  |  |  |
| Non- Squamous | 64/79 (67.4) |  |  |
| Squamous | 31/37 (32.6) | 0.131 | 0.718 |
| Stage |  |  |  |
| IIIB | 16/18 (16.8) |  |  |
| IV | 79/98 (83.2) | 0.703 | 0.402 |
| Smoking history |  |  |  |
| Never | 34/43 (35.8) |  |  |
| Now/Ever | 61/73 (64.2) | 0.368 | 0.544 |
| Distant metastases |  |  |  |
| No | 16/18 (16.8) |  |  |
| Yes | 79/98 (83.2) | 1.070 | 0.301 |
| Driver mutations |  |  |  |
| No | 75/88 (78.9) |  |  |
| Yes | 20/28 (21.1) | 2.728 | 0.099 |
| Radiotherapy |  |  |  |
| No | 40/53 (42.1) |  |  |
| Yes | 55/63 (57.9) | 2.717 | 0.099 |
| Drug regiment |  |  |  |
| 1^st^ line | 44/51 (46.3) |  |  |
| ≥2^nd^ line | 51/65 (53.7) | 1.177 | 0.278 |
| Baseline Total lymphocytes |  |  |  |
| <22.258 | 52/61 (54.7) |  |  |
| ≥22.258 | 43/55 (45.3) | 0.973 | 0.324 |
| Baseline CD3**^+^**T cells |  |  |  |
| <70.950 | 61/77 (64.2) |  |  |
| ≥70.950 | 34/39 (35.8) | 1.106 | 0.293 |
| Baseline CD4**^+^**T cells |  |  |  |
| <39.950 | 57/71 (60.0) |  |  |
| ≥39.950 | 38/45 (40.0) | 0.322 | 0.570 |
| Baseline CD8**^+^**T cells |  |  |  |
| <16.050 | 19/22 (20.0) |  |  |
| ≥16.050 | 76/94 (80.0) | 0.365 | 0.546 |
| Baseline CD4^+^/CD8^+^T cells |  |  |  |
| <1.400 | 42/57 (44.2) |  |  |
| ≥1.400 | 53/59 (55.8) | 5.098 | **0.024** |
| Baseline NK |  |  |  |
| <14.304 | 31/36 (32.6) |  |  |
| ≥14.304 | 64/80 (67.4) | 0.625 | 0.429 |
| Baseline B cells |  |  |  |
| <8.800 | 77/92 (81.1) |  |  |
| ≥8.800 | 18/24 (18.9) | 0.971 | 0.324 |
| Baseline NKT |  |  |  |
| <4.569 | 57/70 (60.0) |  |  |
| ≥4.569 | 38/46 (40.0) | 0.026 | 0.827 |
| Baseline Activated T cells |  |  |  |
| <13.450 | 39/41 (41.1) |  |  |
| ≥13.450 | 56/75 (58.9) | 7.481 | **0.006** |
| Baseline CEA |  |  |  |
| <29.850 | 79/91 (83.2) |  |  |
| ≥29.850 | 16/25 (16.8) | 6.884 | **0.009** |
| Baseline CA125 |  |  |  |
| <74.215 | 70/86 (73.7) |  |  |
| ≥74.215 | 25/30 (26.3) | 0.056 | 0.812 |
| Baseline CA199 |  |  |  |
| <14.000 | 52/59 (54.7) |  |  |
| ≥14.000 | 43/57 (45.3) | 3.152 | 0.076 |
| Baseline NSE |  |  |  |
| <16.470 | 49/62 (51.6) |  |  |
| ≥16.470 | 46/54 (48.4) | 0.737 | 0.391 |
| Δ6W Total lymphocytes |  |  |  |
| Down | 54/61 (56.8) |  |  |
| Up | 41/55 (43.2) | 3.812 | 0.051 |
| Δ6W CD3^+^T cells |  |  |  |
| Down | 42/57 (44.2) |  |  |
| Up | 53/59 (55.8) | 5.098 | **0.024** |
| Δ6W CD4**^+^**T cells |  |  |  |
| Down | 36/47 (37.9) |  |  |
| Up | 59/69 (62.1) | 1.497 | 0.221 |
| Δ6W CD8**^+^**T cells |  |  |  |
| Down | 40/49 (42.1) |  |  |
| Up | 55/67 (57.9) | 0.004 | 0.950 |
| Δ6W CD4**^+^** / CD8**^+^**T cells |  |  |  |
| Down | 43/56 (45.3) |  |  |
| Up | 52/60 (54.7) | 1.907 | 0.167 |
| Δ6W B cells |  |  |  |
| Down | 26/37 (27.4) |  |  |
| Up | 69/79 (72.6) | 4.953 | **0.026** |
| Δ6W NK |  |  |  |
| Down | 59/68 (62.1) |  |  |
| Up | 36/48 (37.9) | 2.627 | 0.105 |
| Δ6W NKT |  |  |  |
| Down | 57/69 (60.0) |  |  |
| Up | 38/47 (40.0) | 0.058 | 0.809 |
| Δ6W Activated T cells |  |  |  |
| Down | 56/64 (58.9) |  |  |
| Up | 39/52 (41.1) | 3.024 | 0.082 |
| Δ6W CEA |  |  |  |
| Down | 45/53 (47.4) |  |  |
| Up | 50/63 (52.6) | 0.596 | 0.440 |
| Δ6W CA125 |  |  |  |
| Down | 41/54 (43.2) |  |  |
| Up | 54/62 (56.8) | 2.429 | 0.119 |
| Δ6W CA199 |  |  |  |
| Down | 49/61 (51.6) |  |  |
| Up | 46/55 (48.4) | 0.214 | 0.644 |
| Δ6W NSE 6W |  |  |  |
| Down | 37/46 (38.9) |  |  |
| Up | 58/70 (61.1) | 0.110 | 0.740 |
| Δ12W Total lymphocytes |  |  |  |
| Down | 58/61 (61.1) |  |  |
| Up | 37/55 (38.9) | 15.086 | **0.000** |
| Δ12W CD3^+^T cells |  |  |  |
| Down | 43/59 (45.3) |  |  |
| Up | 52/57 (54.7) | 6.582 | **0.010** |
| Δ12W CD4**^+^**T cells |  |  |  |
| Down | 34/45 (35.8) |  |  |
| Up | 61/71 (64.2) | 1.994 | 0.158 |
| Δ12W CD8^+^T cells |  |  |  |
| Down | 48/53 (50.5) |  |  |
| Up | 47/63 (49.5) | 4.947 | **0.026** |
| Δ12W CD4^+^ / CD8^+^T cells |  |  |  |
| Down | 42/57 (44.2) |  |  |
| Up | 53/59 (55.8) | 5.098 | **0.024** |
| Δ12W NK |  |  |  |
| Down | 51/60 (53.7) |  |  |
| Up | 44/56 (46.3) | 0.807 | 0.369 |
| Δ12W B cells |  |  |  |
| Down | 32/39 (33.7) |  |  |
| Up | 63/77 (66.3) | 0.001 | 0.975 |
| Δ12W NKT |  |  |  |
| Down | 53/65 (55.8) |  |  |
| Up | 42/51 (44.2) | 0.013 | 0.910 |
| Δ12W Activated T cells |  |  |  |
| Down | 55/66 (57.9) |  |  |
| Up | 40/50 (42.1) | 0.213 | 0.644 |
| Δ12W CEA |  |  |  |
| Down | 44/52 (46.3) |  |  |
| Up | 51/64 (53.7) | 0.470 | 0.493 |
| Δ12W CA199 |  |  |  |
| Down | 50/60 (52.6) |  |  |
| Up | 45/56 (47.4%) | 0.173 | 0.677 |
| Δ12W CA125 |  |  |  |
| Down | 30/42 (31.6) |  |  |
| Up | 65/74 (68.4) | 4.866 | **0.027** |
| Δ12W NSE |  |  |  |
| Down | 32/38 (33.7) |  |  |
| Up | 63/78 (66.3) | 0.204 | 0.651 |

NK, natural killer; NKT, natural killer T; CEA, carcinoembryonic antigen; CA125, carbohydrate antigen 125; CA199, carbohydrate antigen199; NSE, neuron-specific enolase.

**Supplementary Table 6** Multivariable Logistic regression models for DCB

| Parameters | Durable clinical benefit | | |
| --- | --- | --- | --- |
|  | Odds ratio | 95%CI | P value |
| Baseline Activated T cells |  |  |  |
| <13.450 | 1 |  |  |
| ≥13.450 | 0.066 | 0.006-0.782 | 0.031 |
| Baseline CEA |  |  |  |
| <29.850 | 1 |  |  |
| ≥29.850 | 0.106 | 0.018-0.621 | 0.013 |
| Δ12W Total lymphocytes |  |  |  |
| Down | 1 |  |  |
| Up | 13.787 | 2.630-72.271 | 0.002 |
| Δ12W CA125 |  |  |  |
| Down | 1 |  |  |
| Up | 0.160 | 0.032-0.810 | 0.027 |

CEA, carcinoembryonic antigen; CA125, carbohydrate antigen 125.

**Supplementary Table 7** Multivariable Logistic regression of DCB

| Parameters | Durable clinical benefit | | |
| --- | --- | --- | --- |
|  | Odds ratio | 95%CI | P value |
| Baseline CD4**^+^** / CD8**^+^**T cells |  |  |  |
| <1.400 | 1 |  |  |
| ≥1.400 | 1.854 | 0.347-9.901 | 0.470 |
| Δ6W CD3**^+^**T cells |  |  |  |
| Down | 1 |  |  |
| Up | 0.243 | 0.049-1.194 | 0.081 |
| Δ6W B cells |  |  |  |
| Down | 1 |  |  |
| Up | 0.550 | 0.117-2.577 | 0.448 |
| Δ12W CD3**^+^**T cells |  |  |  |
| Down | 1 |  |  |
| Up | 0.204 | 0.030-1.362 | 0.101 |
| Δ12W CD8**^+^**T cells |  |  |  |
| Down | 1 |  |  |
| Up | 1.168 | 0.200-6.819 | 0.863 |
| Δ12W CD4**^+^** / CD8**^+^**T cells |  |  |  |
| Down | 1 |  |  |
| Up | 0.407 | 0.064-2.607 | 0.343 |

**Supplementary Table 8** Univariable and Multivariable Cox regression analysis of Progression free survival

| Parameters | Univariable | | |  | Multivariable | | |
| --- | --- | --- | --- | --- | --- | --- | --- |
|  | HR | 95%CI | P value |  | HR | 95%CI | P value |
| ECOG PS |  |  |  |  |  |  |  |
| 0  1  2 | 1  2.451  8.885 | 1.052-5.711  3.119-25.307 | 0.038*  0.000* |  | 1  3.117  9.246 | 1.313-7.403  3.091-27.655 | 0.010*  0.000* |
| Baseline CD8^+^T cells |  |  |  |  |  |  |  |
| <16.050 | 1 |  |  |  | 1 |  |  |
| ≥16.050 | 2.187 | 1.077 – 4.440 | 0.030* |  | 1.881 | 0.865-4.087 | 0.111 |
| Baseline CD4^+^ / CD8^+^T cells |  |  |  |  |  |  |  |
| <1.400 | 1 |  |  |  | 1 |  |  |
| ≥1.400 | 0.497 | 0.306 - 0.807 | 0.005* |  | 0.543 | 0.326-0.906 | 0.019* |
| Baseline Activated T cells |  |  |  |  |  |  |  |
| <13.450 | 1 |  |  |  | 1 |  |  |
| ≥13.450 | 2.334 | 1.344 – 4.052 | 0.003* |  | 2.151 | 1.207-3.836 | 0.009* |
| Baseline CA125 |  |  |  |  |  |  |  |
| <74.215 | 1 |  |  |  | 1 |  |  |
| ≥74.215 | 0.411 | 0.214-0.787 | 0.007* |  | 0.401 | 0.197-0.816 | 0.012* |
| Δ12W Total lymphocytes |  |  |  |  |  |  |  |
| Down | 1 |  |  |  | 1 |  |  |
| Up | 0.457 | 0.282 - 0.740 | 0.001* |  | 0.372 | 0.223-0.619 | 0.000* |
| Δ12W CA125 |  |  |  |  |  |  |  |
| Down | 1 |  |  |  | 1 |  |  |
| Up | 1.759 | 1.086 - 2.851 | 0.022* |  | 1.736 | 1.042-2.892 | 0.034* |

*, P<0.05. CA125, carbohydrate antigen 125

**Supplementary Table 9** Univariable and Multivariable Cox regression analysis of Overall survival

| Parameters | Univariable | | |  | Multivariable | | |
| --- | --- | --- | --- | --- | --- | --- | --- |
|  | HR | 95%CI | P value |  | HR | 95%CI | P value |
| Baseline Activated T cells |  |  |  |  |  |  |  |
| <13.450 | 1 |  |  |  | 1 |  |  |
| ≥13.450 | 3.160 | 1.195-8.358 | 0.020* |  | 3.240 | 1.152 – 9.114 | 0.026* |
| Δ6w CD3^+^T cells |  |  |  |  |  |  |  |
| Down | 1 |  |  |  | 1 |  |  |
| Up | 3.640 | 1.546-8.568 | 0.003* |  | 3.163 | 1.267 – 7.900 | 0.014* |
| Δ6w CD4^+^T cells |  |  |  |  |  |  |  |
| Down | 1 |  |  |  | 1 |  |  |
| Up | 2.544 | 1.174-5.512 | 0.018* |  | 1.639 | 0.696 – 3.860 | 0.259 |
| Δ6w Activated T cells |  |  |  |  |  |  |  |
| Down | 1 |  |  |  | 1 |  |  |
| Up | 0.418 | 0.191-0.915 | 0.029* |  | 0.608 | 0.263 - 1.404 | 0.244 |

*, P<0.05.

**Supplementary Table 10** Median months of PFS and OS in each group

| Parameters | Median PFS | | | | | Median OS | | | | |
| --- | --- | --- | --- | --- | --- | --- | --- | --- | --- | --- |
|  | Δ6W | | Δ12W | | | Δ6W | | Δ12W | | |
|  | Median, months | P value | Median, months | | P value | Median, months | P value | Median, months | | P value |
| Total lymphocytes |  | |  | | |  | |  | | |
| Up | 20 | 0.109 | 23.3 | 0.001 | | - | 0.860 | - | 0.054 | |
| Down | 13.9 |  | 11.5 |  | | - |  | 32.7 |  | |
| CD3^+^T cells |  | |  | | |  | |  | | |
| Up | 17.9 | 0.308 | 14.2 | 0.142 | | 32.7 | 0.002 | - | 0.848 | |
| Down | 12.8 |  | 17.9 |  | | - |  | - |  | |
| CD4^+^T cells |  | |  | | |  | |  | | |
| Up | 12.7 | 0.499 | 15.4 | 0.829 | | 32.7 | 0.014 | - | 0.502 | |
| Down | 17.4 |  | 13.9 |  | | - |  | - |  | |
| CD8^+^T cells |  | |  | | |  | |  | | |
| Up | 13.9 | 0.652 | 20 | 0.101 | | - | 0.200 | - | 0.528 | |
| Down | 17.4 |  | 12.6 |  | | - |  | 32.7 |  | |
| CD4^+^ / CD8^+^T cells |  | |  | | |  | |  | | |
| Up | 20.4 | 0.080 | 14.2 | 0.110 | | - | 0.850 | - | 0.866 | |
| Down | 12.7 |  | 17.4 |  | | - |  | - |  | |
| NK |  | |  | | |  | |  | | |
| Up | 14.2 | 0.447 | 12.6 | 0.712 | | - | 0.807 | - | 0.685 | |
| Down | 15.4 |  | 17.9 |  | | - |  | - |  | |
| B cells |  | |  | | |  | |  | | |
| Up | 14.6 | 0.650 | 18.8 | 0.393 | | - | 0.745 | - | 0.886 | |
| Down | 12.8 |  | 13.9 |  | | - |  | - |  | |
| NKT |  | |  | | |  | |  | | |
| Up | 17.9 | 0.650 | 14.2 | 0.176 | | - | 0.498 | - | 0.954 | |
| Down | 14.2 |  | 18.8 |  | | - |  | - |  | |
| Activated T cells |  | |  | | |  | |  | | |
| Up | 12.8 | 0.170 | 14.3 | 0.864 | | - | 0.024 | - | 0.188 | |
| Down | 15.4 |  | 15.4 |  | | 32.7 |  | - |  | |
| CEA |  |  |  | | |  |  |  | | |
| Up | 12.6 | 0.517 | 14.2 | 0.598 | | - | 0.570 | - | 0.883 | |
| Down | 17.4 |  | 17.4 |  | | - |  | - |  | |
| CA125 |  |  |  | | |  |  |  | | |
| Up | 12.6 | 0.073 | 11.5 | 0.020 | | - | 0.180 | - | 0.231 | |
| Down | 20.4 |  | 20 |  | | - |  | - |  | |
| CA199 |  |  |  | | |  |  |  | | |
| Up | 17.4 | 0.736 | 15.4 | 0.857 | | 32.7 | 0.576 | - | 0.982 | |
| Down | 14.2 |  | 12.7 |  | | - |  | - |  | |
| NSE |  |  |  | | |  |  |  | | |
| Up | 11.6 | 0.119 | 14.2 | 0.421 | | - | 0.804 | 32.7 | 0.150 | |
| Down | 17.4 |  | 14.6 |  | | - |  | - |  | |

**-**:NR. NK, natural killer; NKT, natural killer T; CEA, carcinoembryonic antigen; CA125, carbohydrate antigen 125; CA199, carbohydrate antigen199; NSE, neuron-specific enolase.

**Supplementary Table 11** Univariable Cox regression of Progression free survival

| Parameters | Progression free survival | | |
| --- | --- | --- | --- |
|  | HR | 95%CI | P value |
| Age |  |  |  |
| <63 | 1 |  |  |
| ≥63 | 0.941 | 0.581-1.525 | 0.806 |
| Gender |  |  |  |
| Female | 1 |  |  |
| Male | 1.369 | 0.805-2.329 | 0.247 |
| Histology |  |  |  |
| Non- Squamous | 1 |  |  |
| Squamous | 0.989 | 0.591-1.655 | 0.965 |
| Stage |  |  |  |
| IIIB | 1 |  |  |
| IV | 1.081 | 0.535-2.184 | 0.829 |
| Differentiation |  |  |  |
| Moderate | 1 |  |  |
| Medium-Low | 0.604 | 0.218-1.675 | 0.333 |
| Low | 0.789 | 0.316-1.972 | 0.612 |
| NA | 0.463 | 0.192-1.117 | 0.087 |
| Smoking history |  |  |  |
| Never | 1 |  |  |
| Now/Ever | 0.751 | 0.464-1.215 | 0.243 |
| Distant metastases |  |  |  |
| No | 1 |  |  |
| Yes | 1.375 | 0.682-2.775 | 0.373 |
| Driver mutations |  |  |  |
| No | 1 |  |  |
| Yes | 1.230 | 0.709-2.133 | 0.461 |
| PD-1 inhibitor type |  |  |  |
| Pembrolizumab | 1 |  |  |
| Toripalimab | 1.210 | 0.585-2.504 | 0.608 |
| Camrelizumab | 1.338 | 0.701-2.556 | 0.377 |
| Sintilimab | 0.809 | 0.441-1.484 | 0.493 |
| Combination regimen |  |  |  |
| Monotherapy | 1 |  |  |
| Chemotherapy | 0.660 | 0.205-2.130 | 0.487 |
| Anti-angiogenic therapy | 0.879 | 0.220-3.520 | 0.855 |
| Both | 0.415 | 0.107-1.614 | 0.205 |
| Drug regimen |  |  |  |
| 1^st^ line | 1 |  |  |
| ≥2^nd^ line | 1.309 | 0.805-2.129 | 0.277 |
| Radiotherapy |  |  |  |
| No | 1 |  |  |
| Yes | 0.843 | 0.523-1.358 | 0.482 |
| Baseline Total lymphocytes |  |  |  |
| <22.258 | 1 |  |  |
| ≥22.258 | 1.489 | 0.923-2.402 | 0.102 |
| Baseline CD3^+^T cells |  |  |  |
| <70.950 | 1 |  |  |
| ≥70.950 | 0.584 | 0.337-1.012 | 0.055 |
| Baseline CD4^+^T cells |  |  |  |
| <39.950 | 1 |  |  |
| ≥39.950 | 0.608 | 0.361-1.024 | 0.061 |
| Baseline NK |  |  |  |
| <14.304 | 1 |  |  |
| ≥14.304 | 1.366 | 0.797-2.342 | 0.257 |
| Baseline B cells |  |  |  |
| <8.800 | 1 |  |  |
| ≥8.800 | 1.444 | 0.833-2.501 | 0.190 |
| Baseline NKT |  |  |  |
| <4.569 | 1 |  |  |
| ≥4.569 | 1.207 | 0.748-1.946 | 0.441 |
| Baseline CEA |  |  |  |
| <29.850 | 1 |  |  |
| ≥29.850 | 1.265 | 0.720-2.224 | 0.413 |
| Baseline CA199 |  |  |  |
| <14.000 | 1 |  |  |
| ≥14.000 | 1.586 | 0.980-2.565 | 0.060 |
| Baseline NSE |  |  |  |
| <16.470 | 1 |  |  |
| ≥16.470 | 0.633 | 0.388-1.032 | 0.066 |
| Δ6W Total lymphocytes |  |  |  |
| Down | 1 |  |  |
| Up | 0.680 | 0.422-1.095 | 0.112 |
| Δ6W CD3^+^T cells |  |  |  |
| Down | 1 |  |  |
| Up | 1.280 | 0.794-2.062 | 0.311 |
| Δ6W CD4^+^T cells |  |  |  |
| Down | 1 |  |  |
| Up | 1.180 | 0.729-1.909 | 0.500 |
| Δ6W CD8^+^T cells |  |  |  |
| Down | 1 |  |  |
| Up | 0.895 | 0.552-1.452 | 0.653 |
| Δ6W CD4^+^ / CD8^+^T cells |  |  |  |
| Down | 1 |  |  |
| Up | 1.534 | 0.945-2.490 | 0.083 |
| Δ6W NK |  |  |  |
| Down | 1 |  |  |
| Up | 0.830 | 0.513-1.344 | 0.449 |
| Δ6W B cells |  |  |  |
| Down | 1 |  |  |
| Up | 1.125 | 0.676-1.870 | 0.651 |
| Δ6W NKT |  |  |  |
| Down | 1 |  |  |
| Up | 1.119 | 0.688-1.821 | 0.651 |
| Δ6W Activated T cells |  |  |  |
| Down | 1 |  |  |
| Up | 0.718 | 0.446-1.156 | 0.173 |
| Δ6W CEA |  |  |  |
| Down | 1 |  |  |
| Up | 1.170 | 0.726-1.887 | 0.518 |
| Δ6W CA125 |  |  |  |
| Down | 1 |  |  |
| Up | 1.542 | 0.956-2.485 | 0.076 |
| Δ6W CA199 |  |  |  |
| Down | 1 |  |  |
| Up | 1.085 | 0.674-1.748 | 0.736 |
| Δ6W NSE |  |  |  |
| Down | 1 |  |  |
| Up | 1.459 | 0.904-2.355 | 0.122 |
| Δ12W CD3^+^T cells |  |  |  |
| Down | 1 |  |  |
| Up | 1.432 | 0.884-2.320 | 0.145 |
| Δ12W CD4^+^T cells |  |  |  |
| Down | 1 |  |  |
| Up | 1.055 | 0.650-1.712 | 0.829 |
| Δ12W CD8^+^T cells |  |  |  |
| Down | 1 |  |  |
| Up | 0.668 | 0.411-1.087 | 0.105 |
| Δ12W CD4^+^ / CD8^+^T cells |  |  |  |
| Down | 1 |  |  |
| Up | 1.476 | 0.912-2.390 | 0.113 |
| Δ12W NK |  |  |  |
| Down | 1 |  |  |
| Up | 1.094 | 0.679-1.763 | 0.713 |
| Δ12W B cells |  |  |  |
| Down | 1 |  |  |
| Up | 0.802 | 0.482-1.334 | 0.395 |
| Δ12W NKT |  |  |  |
| Down | 1 |  |  |
| Up | 1.400 | 0.857-2.286 | 0.179 |
| Δ12W Activated T cells |  |  |  |
| Down | 1 |  |  |
| Up | 0.959 | 0.594-1.549 | 0.864 |
| Δ12W CEA |  |  |  |
| Down | 1 |  |  |
| Up | 1.137 | 0.704-1.836 | 0.599 |
| Δ12W CA199 |  |  |  |
| Down | 1 |  |  |
| Up | 0.957 | 0.595-1.540 | 0.857 |
| Δ12W NSE |  |  |  |
| Down | 1 |  |  |
| Up | 1.224 | 0.746-2.008 | 0.423 |

NK, natural killer; NKT, natural killer T; CEA, carcinoembryonic antigen; CA125, carbohydrate antigen 125; CA199, carbohydrate antigen199; NSE, neuron-specific enolase.

**Supplementary Table 12** Univariable Cox regression of Overall survival

| Parameters | Overall survival | | |
| --- | --- | --- | --- |
|  | HR | 95%CI | P value |
| Age |  |  |  |
| <63 | 1 |  |  |
| ≥63 | 0.689 | 0.323-1.472 | 0.337 |
| Gender |  |  |  |
| Female | 1 |  |  |
| Male | 1.651 | 0.741-3.679 | 0.220 |
| Histology |  |  |  |
| Non- Squamous | 1 |  |  |
| Squamous | 1.217 | 0.544-2.724 | 0.632 |
| Stage |  |  |  |
| IIIB | 1 |  |  |
| IV | 0.926 | 0.319-2.685 | 0.887 |
| Differentiation |  |  |  |
| Moderate | 1 |  |  |
| Medium-Low | 1.501 | 0.290-7.758 | 0.628 |
| Low | 1.226 | 0.253-5.940 | 0.800 |
| NA | 0.994 | 0.223-4.437 | 0.994 |
| ECOG PS |  |  |  |
| 0 | 1 |  |  |
| 1 | 2.898 | 0.683-12.298 | 0.149 |
| 2 | 0.368 | 0.368-18.640 | 0.336 |
| Smoking history |  |  |  |
| Never | 1 |  |  |
| Now/Ever | 0.905 | 0.420-1.952 | 0.799 |
| Distant metastases |  |  |  |
| No | 1 |  |  |
| Yes | 1.655 | 0.498-5.505 | 0.411 |
| Driver mutations |  |  |  |
| No | 1 |  |  |
| Yes | 1.458 | 0.637-3.334 | 0.372 |
| PD-1 inhibitor type |  |  |  |
| Pembrolizumab | 1 |  |  |
| Toripalimab | 1.291 | 0.396-4.203 | 0.672 |
| Camrelizumab | 1.002 | 0.308-3.259 | 0.998 |
| Sintilimab | 1.544 | 0.626-3.810 | 0.346 |
| Drug regimen |  |  |  |
| 1^st^ line | 1 |  |  |
| ≥2^nd^ line | 1.477 | 0.662-3.297 | 0.341 |
| Radiotherapy |  |  |  |
| No | 1 |  |  |
| Yes | 0.525 | 0.243-1.134 | 0.101 |
| Baseline Total lymphocytes |  |  |  |
| <22.258 | 1 |  |  |
| ≥22.258 | 1.123 | 0.526-2.398 | 0.764 |
| Baseline CD3^+^T cells |  |  |  |
| <70.950 | 1 |  |  |
| ≥70.950 | 0.922 | 0.403-2.109 | 0.848 |
| Baseline CD4^+^T cells |  |  |  |
| <39.950 | 1 |  |  |
| ≥39.950 | 1.192 | 0.553-2.572 | 0.654 |
| Baseline CD8^+^T cells |  |  |  |
| <16.050 | 1 |  |  |
| ≥16.050 | 1.474 | 0.556-3.911 | 0.435 |
| Baseline CD4^+^ / CD8^+^T cells |  |  |  |
| <1.400 | 1 |  |  |
| ≥1.400 | 0.719 | 0.336-1.537 | 0.394 |
| Baseline NK |  |  |  |
| <14.304 | 1 |  |  |
| ≥14.304 | 1.161 | 0.490-2.751 | 0.734 |
| Baseline B cells |  |  |  |
| <8.800 | 1 |  |  |
| ≥8.800 | 0.757 | 0.286-2.003 | 0.574 |
| Baseline NKT |  |  |  |
| <4.569 | 1 |  |  |
| ≥4.569 | 1.125 | 0.526-2.407 | 0.761 |
| Baseline CEA |  |  |  |
| <29.850 | 1 |  |  |
| ≥29.850 | 1.261 | 0.532-2.985 | 0.598 |
| Baseline CA199 |  |  |  |
| <14.000 | 1 |  |  |
| ≥14.000 | 0.612 | 0.283-1.325 | 0.213 |
| Baseline CA125 |  |  |  |
| <74.215 | 1 |  |  |
| ≥74.215 | 0.336 | 0.101-1.118 | 0.075 |
| Baseline NSE |  |  |  |
| <16.470 | 1 |  |  |
| ≥16.470 | 0.582 | 0.266-1.273 | 0.176 |
| Δ6W Total lymphocytes |  |  |  |
| Down | 1 |  |  |
| Up | 0.934 | 0.439-1.989 | 0.860 |
| Δ6W CD8^+^T cells |  |  |  |
| Down | 1 |  |  |
| Up | 1.636 | 0.765-3.502 | 0.205 |
| Δ6W CD4^+^ / CD8^+^T cells |  |  |  |
| Down | 1 |  |  |
| Up | 1.076 | 0.503-2.304 | 0.850 |
| Δ6W NK |  |  |  |
| Down | 1 |  |  |
| Up | 0.908 | 0.420-1.965 | 0.807 |
| Δ6W B cells |  |  |  |
| Down | 1 |  |  |
| Up | 1.143 | 0.512-2.552 | 0.745 |
| Δ6W NKT |  |  |  |
| Down | 1 |  |  |
| Up | 1.318 | 0.591-2.937 | 0.499 |
| Δ6W CEA |  |  |  |
| Down | 1 |  |  |
| Up | 1.244 | 0.584-2.650 | 0.571 |
| Δ6W CA125 |  |  |  |
| Down | 1 |  |  |
| Up | 1.682 | 0.779-3.631 | 0.186 |
| Δ6W CA199 |  |  |  |
| Down | 1 |  |  |
| Up | 1.242 | 0.580-2.658 | 0.577 |
| Δ6W NSE |  |  |  |
| Down | 1 |  |  |
| Up | 1.103 | 0.508-2.393 | 0.804 |
| Δ12W Total lymphocytes |  |  |  |
| Down | 1 |  |  |
| Up | 0.472 | 0.216-1.032 | 0.060 |
| Δ12W CD3^+^T cells |  |  |  |
| Down | 1 |  |  |
| Up | 0.929 | 0.435-1.983 | 0.848 |
| Δ12W CD4^+^T cells |  |  |  |
| Down | 1 |  |  |
| Up | 1.294 | 0.608-2.755 | 0.504 |
| Δ12W CD8^+^T cells |  |  |  |
| Down | 1 |  |  |
| Up | 0.782 | 0.363-1.683 | 0.529 |
| Δ12W CD4^+^ / CD8^+^T cells |  |  |  |
| Down | 1 |  |  |
| Up | 1.067 | 0.500-2.280 | 0.866 |
| Δ12W NK |  |  |  |
| Down | 1 |  |  |
| Up | 1.169 | 0.549-2.488 | 0.686 |
| Δ12W B cells |  |  |  |
| Down | 1 |  |  |
| Up | 0.943 | 0.422-2.106 | 0.886 |
| Δ12W NKT |  |  |  |
| Down | 1 |  |  |
| Up | 0.978 | 0.457-2.091 | 0.954 |
| Δ12W Activated T cells |  |  |  |
| Down | 1 |  |  |
| Up | 0.603 | 0.282-1.289 | 0.192 |
| Δ12W CEA |  |  |  |
| Down | 1 |  |  |
| Up | 0.945 | 0.441-2.023 | 0.884 |
| Δ12W CA199 |  |  |  |
| Down | 1 |  |  |
| Up | 1.009 | 0.474-2.148 | 0.982 |
| Δ12W CA125 |  |  |  |
| Down | 1 |  |  |
| Up | 1.587 | 0.740-3.403 | 0.236 |
| Δ12W NSE |  |  |  |
| Down | 1 |  |  |
| Up | 1.735 | 0.811-3.710 | 0.155 |

NK, natural killer; NKT, natural killer T; CEA, carcinoembryonic antigen; CA125, carbohydrate antigen 125; CA199, carbohydrate antigen199; NSE, neuron-specific enolase.
